# Intestinal epithelial MyD88 is a sensor switching host metabolism towards obesity according to nutritional status

**DOI:** 10.1038/ncomms6648

**Published:** 2014-12-05

**Authors:** Amandine Everard, Lucie Geurts, Robert Caesar, Matthias Van Hul, Sébastien Matamoros, Thibaut Duparc, Raphael G. P. Denis, Perrine Cochez, Florian Pierard, Julien Castel, Laure B. Bindels, Hubert Plovier, Sylvie Robine, Giulio G. Muccioli, Jean-Christophe Renauld, Laure Dumoutier, Nathalie M. Delzenne, Serge Luquet, Fredrik Bäckhed, Patrice D. Cani

**Affiliations:** 1 Université Catholique de Louvain, Louvain Drug Research Institute, WELBIO (Walloon Excellence in Life sciences and BIOtechnology), Metabolism and Nutrition Research Group, B-1200 Brussels, Belgium; 2 Wallenberg Laboratory/Sahlgrenska Center for Cardiovascular and Metabolic Research, Sahlgrenska University Hospital, Gothenburg 40530, Sweden; 3 Department of Molecular and Clinical Medicine, University of Gothenburg, Gothenburg 41345, Sweden; 4 Université Paris Diderot, Sorbonne Paris Cité, BFA, UMR8251, CNRS, F-75205 Paris, France; 5 Université Catholique de Louvain, Ludwig Institute for Cancer Research, Experimental Medicine Unit, B-1200 Brussels, Belgium; 6 Institut Curie, Centre National de la Recherche Scientifique, Unité Mixte de Recherche 144, F-75005 Paris, France; 7 Université Catholique de Louvain, Louvain Drug Research Institute, Bioanalysis and Pharmacology of Bioactive Lipids Research Group, B-1200 Brussels, Belgium

## Abstract

Obesity is associated with a cluster of metabolic disorders, low-grade inflammation and altered gut microbiota. Whether host metabolism is controlled by intestinal innate immune system and the gut microbiota is unknown. Here we report that inducible intestinal epithelial cell-specific deletion of MyD88 partially protects against diet-induced obesity, diabetes and inflammation. This is associated with increased energy expenditure, an improved glucose homeostasis, reduced hepatic steatosis, fat mass and inflammation. Protection is transferred following gut microbiota transplantation to germ-free recipients. We also demonstrate that intestinal epithelial MyD88 deletion increases anti-inflammatory endocannabinoids, restores antimicrobial peptides production and increases intestinal regulatory T cells during diet-induced obesity. Targeting MyD88 after the onset of obesity reduces fat mass and inflammation. Our work thus identifies intestinal epithelial MyD88 as a sensor changing host metabolism according to the nutritional status and we show that targeting intestinal epithelial MyD88 constitutes a putative therapeutic target for obesity and related disorders.

## Abstract (web summary)

Gut microbes are known to influence whole-body metabolism. Here Everard *et al.* show the adaptor protein MyD88 in intestinal epithelial cells is sensitive to changes in the diet and affects composition of the gut microbiota, which influences the development of obesity and associated diseases.

## Graphical abstract

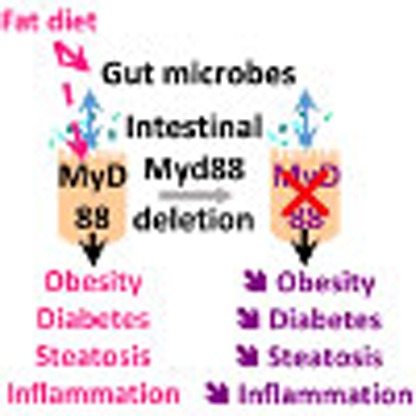

The increasing burden of obesity is alarming because of its broad spectrum of acute and long-term complications (for example, type 2 diabetes, cardiovascular diseases and non-alcoholic fatty liver disease). A large body of evidence suggests that the gut microbiota is altered in obesity and type 2 diabetes^1^,^2^,^3^,^4^. Moreover, we and others have provided data showing that gut microbes influence whole-body metabolism by affecting energy balance but also inflammation and gut barrier function; however, the mechanisms are unclear and are likely multifaceted^5^,^6^,^7^. The intestinal epithelium is the first organ in contact with ingested food and nutrients. This is also the largest surface of cross-talks with the outside part of the body, including with gut microbes. The intestinal innate immune system is one of the most important factors involved in the interactions between gut microbiota and the host. This symbiosis may occur through a fine-tuned mechanism leading to pathogen destruction and, conversely, to the tolerance of commensals by selecting strategies to create ecological niches for beneficial and stably associated gut microbiota^8^,^9^. Recognition of pathogen-associated molecular patterns by epithelial cells through several effector systems (that is, pathogen recognition receptors) is important for this balance. MyD88 (myeloid differentiation primary response gene 88) is a central adaptor molecule for the majority of Toll-like receptors (TLRs), which are the most studied pathogen recognition receptors. MyD88 is a protein that is at the interface of the interaction between microorganisms and the host. Previous studies using whole-body deletion of specific TLRs^10^,^11^ or MyD88 support a key role of the immune system in shaping the gut microbiota and in energy homeostasis^12^,^13^,^14^. However, numerous conflicting reports exist about the protection against diet-induced obesity and associated disorders^12^,^13^,^15^. Intestinal epithelial cells are in constant interaction with intestinal luminal content and can contribute to host defence through several mechanisms. However, it is at present unknown whether the epithelial MyD88-dependent signalling is involved in mediating these effects. Here we examined the possibility that MyD88 in the intestinal epithelial cell acts as a sensor involved in the interaction between nutrients, gut microbes and the host in obesity. Our data suggest that intestinal epithelial MyD88 is a sensor changing host metabolism according to the diet and influences the composition of the gut microbiota thereby influencing energy metabolism and the development of obesity and associated diseases. Thus, we show that targeting intestinal epithelial MyD88 is a putative therapeutic target for obesity and related disorders.

## Results

### Intestinal epithelial cells MyD88 controls energy storage during obesity

**Figure 1:**
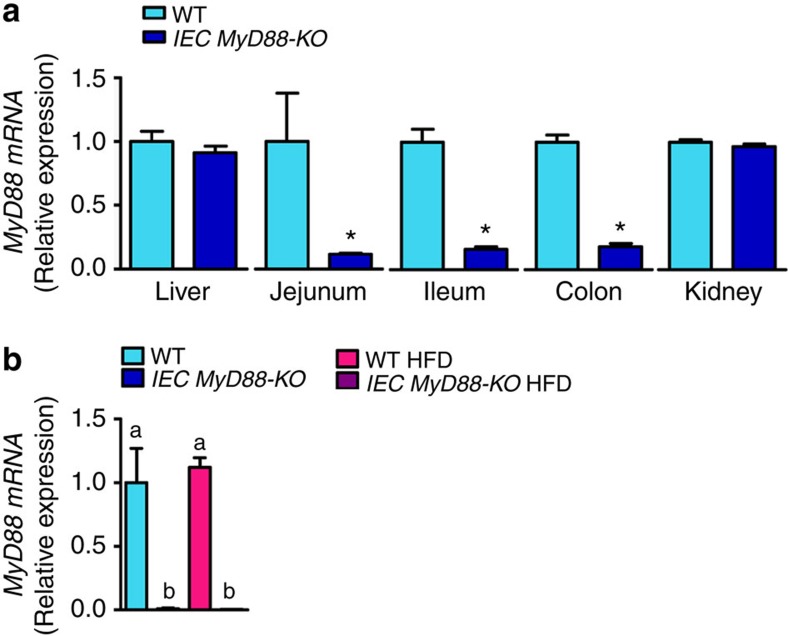
Validation of intestinal specific deletion of MyD88. (**a**) The tamoxifen induction of the intestinal epithelial MyD88 deletion in adult mice (8-week-old mice) is associated with a drastic decrease in *MyD88* mRNA expression, specifically in the intestine. mRNA expression of *MyD88* in the liver, jejunum, ileum, colon and kidney (*n*=7). (**b**) The residual expression of MyD88 corresponds to the other cell types than intestinal epithelial cells since the expression of MyD88 was reduced to almost undetectable values in isolated epithelial cells from *IEC MyD88 KO* mice (*n*=4). These data correspond to the results of one experiment. All the replicates represent biological replicates. Data are shown as the means±s.e.m. Data with * are significantly different (*P<*0.05) according to the unpaired two-tailed Student’s *t*-test. Data with different superscript letters are significantly different (*P<*0.05) according to the one-way analysis of variance statistical analysis followed by Newman–Keuls *post hoc* tests after normalization by log transformation.

**Figure 2:**
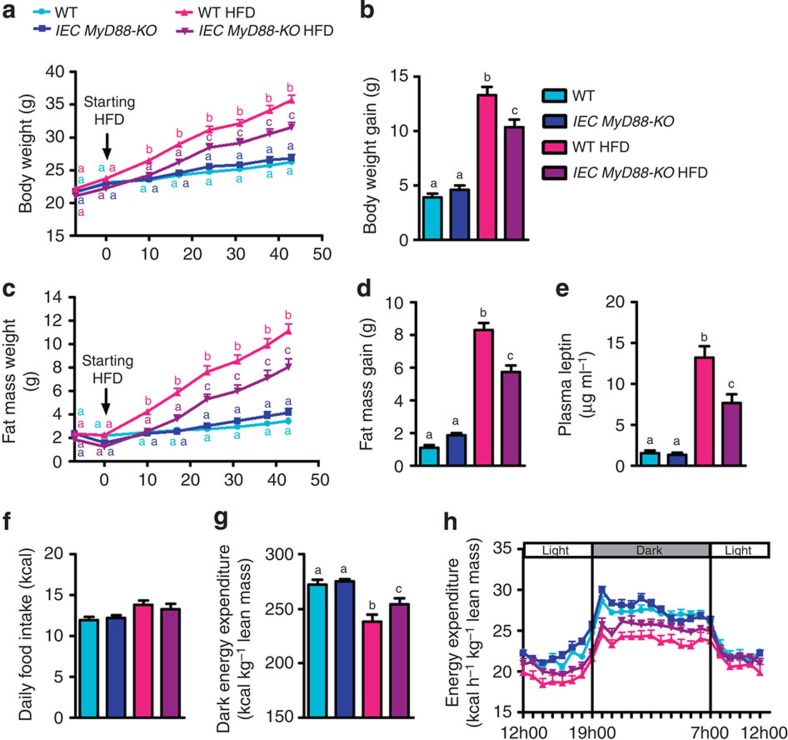
*IEC MyD88-KO* mice are partially protected against HFD-induced obesity. Mice were monitored during 8 weeks of a HFD or a control diet. (**a**) Body weight evolution over 6 weeks of treatment (g; *n*=25). (**b**) Total body weight gain (g; *n*=25). (**c**) Fat mass weight evolution over 6 weeks of treatment (g; *n*=25). (**d**) Total fat mass gain (g; *n*=25). These data (**a**–**d**) correspond to the results of three independent experiments. (**e**) Leptin plasma levels (*n*=10). (**f**) Daily (24 h) food intake precisely measured in metabolic chambers (kcal; *n*=6). (**g**) Dark (night) energy expenditure (kcal) normalized to total lean mass (kg; *n*=6). (**h**) Light and dark cycle energy expenditure (kcal) normalized to total lean mass (kg) measured in metabolic chambers during indirect calorimetry studies (*n*=6). These data (**e**–**h**) correspond to the results of one experiment. All the replicates represent biological replicates. Data are shown as the means±s.e.m. Data with different superscript letters are significantly different (*P<*0.05) according to the one-way analysis of variance statistical analysis followed by Newman–Keuls *post hoc* tests after normalization by log transformation (**b**,**d**–**g**) or two-way statistical analysis followed by Bonferroni *post hoc* test (**a** and **c**).

**Figure 3:**
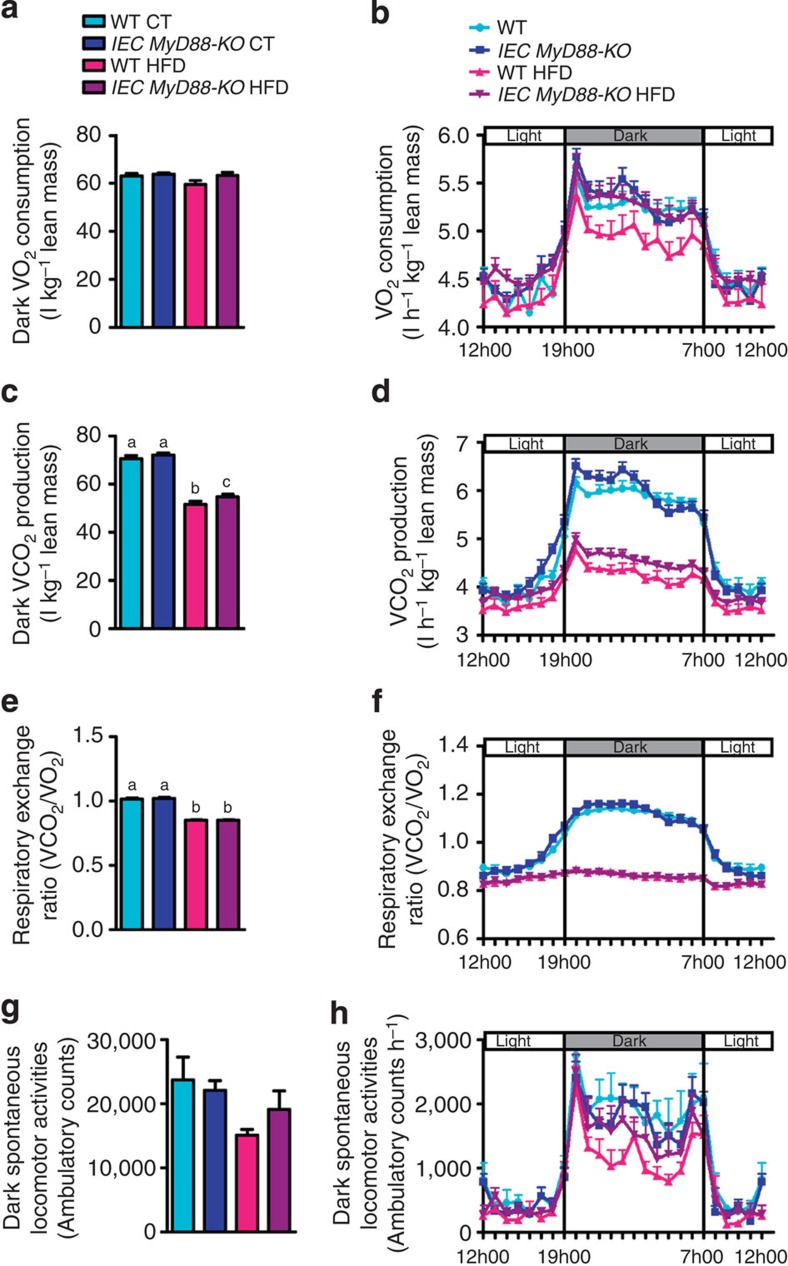
IEC MyD88 deletion increases CO_2_ production and O_2_ consumption under HFD. (**a**) Dark (night) O_2_ consumption (ml) normalized to total lean mass (kg; *n*=6). (**b**) Light and dark cycle of O_2_ consumption (ml) normalized to total lean mass (kg; *n*=6). (**c**) Dark (night) CO_2_ production (ml) normalized to total lean mass (kg; *n*=6). (**d**) Light and dark cycle of CO_2_ production (ml) normalized to total lean mass (kg; *n*=6). (**e**) Respiratory exchange ratio (*n*=6). (**f**) Light and dark cycle of respiratory exchange ratio (*n*=6). (**g**) Dark ambulatory activities (counts; *n*=6). (**h**) Light and dark cycle of ambulatory activities (counts; *n*=6). These data correspond to the results of one experiment. All the replicates represent biological replicates. Data are shown as the means±s.e.m. (**a**,**c**,**e**,**g**) Data with different superscript letters are significantly different (*P<*0.05) according to the one-way analysis of variance statistical analysis followed by Newman–Keuls *post hoc* tests after normalization by log transformation (**a**,**c**,**e** and **g**).

We generated mice with an inducible intestinal epithelial cell (IEC)-specific deletion of MyD88 (*IEC MyD88-KO*; Fig. 1). The intestinal MyD88 deletion is induced by a tamoxifen injection in adult animals before starting the dietary intervention (Fig. 1). We found that following a chronic high-fat diet (HFD) challenge, *IEC MyD88-KO* mice gained less weight than wild-type (WT) mice throughout the 8 weeks of the HFD treatment. IEC MyD88 deletion reduced body weight compared with WT-HFD mice. It is worth noting that the intestinal MyD88 deletion did not affect the body weight in control diet (CT)-fed mice (Fig. 2a,b). Body composition analysis using nuclear magnetic resonance (NMR) revealed that *IEC MyD88-KO* HFD mice gained less fat mass throughout the experiment. Whole-body fat mass was 30% lower in *IEC MyD88-KO* HFD mice than in WT HFD mice (Fig. 2c,d), and this reduction was associated with a dramatic decrease in plasma leptin, an adipokine produced proportionally to fat mass (Fig. 2e). We also noticed that the different fat depots were similarly affected by IEC MyD88 deletion (Supplementary Fig. 1c–e). These results suggest that in addition to reducing body weight, the IEC MyD88 deletion decreased the fat mass in favour of lean mass (Supplementary Fig. 1a,b). The reduced body weight and fat mass were independent of changes in food intake (Fig. 2f). This phenotype was also independent of changes in nutrient intestinal absorption, as we did not find any changes in the calories remaining in the feces of the mice (WT HFD: 3472±35.24 and *IEC MyD88-KO* HFD: 3471±34.75; feces energy content (cal g^−1^ of feces)). We next analysed the energy efficiency by using indirect calorimetry in *IEC MyD88-KO* mice. We found that under basal conditions (CT), IEC MyD88 deletion did not affect energy expenditure. However, during HFD feeding, *IEC MyD88-KO* mice exhibited increased energy expenditure and CO_2_ production (Figs 2g,h and 3c,d) and a trend to increase O_2_ consumption (Fig. 3a,b) compared with WT mice during the dark phase, without affecting the respiratory exchange ratio (Fig. 3e,f). The increased energy expenditure was partly due to a trend to increase spontaneous locomotor activities (Fig. 3g,h). These data suggest that physical activity contributes to the reduction in body weight and fat mass development in *IEC MyD88-KO* mice during HFD feeding.

### Glucose metabolism and inflammation in *IEC MyD88-KO* mice

**Figure 4:**
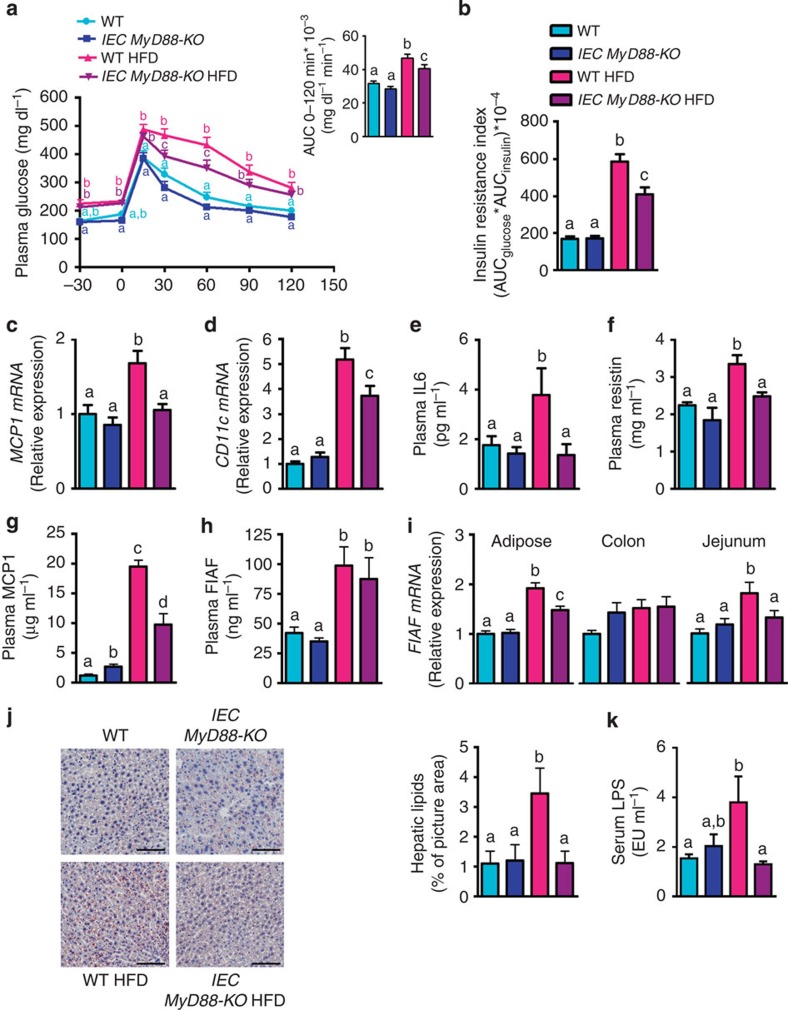
IEC MyD88 deletion improves metabolic disorders associated with obesity. (**a**) Oral glucose tolerance test. Inset: area under the curve (AUC) during oral glucose tolerance test (*n*=10). (**b**) Insulin resistance index (AUC blood glucose × AUC insulin) (*n*=10). (**c**,**d**) *MCP1* mRNA and *CD11c* mRNA in the adipose tissue (*n*=25). (**e**) Circulating IL6 and (**f**) Resistin, (**g**) MCP1 and (**h**) FIAF plasma levels (*n*=10). (**i**) *FIAF* mRNA in the adipose tissue, the colon and the jejunum (*n*=25). (**j**) Liver oil red O staining, scale bar, 100 μm. (**k**) Serum LPS levels measured in the portal vein (EU ml^−1^) (*n*=6). Data from **c** to **d** correspond to the results of three independent experiments. The data (**a**,**b** and **e**–**k**) correspond to the results of one experiment. All the replicates represent biological replicates. Data are shown as the means±s.e.m. Data with different superscript letters are significantly different (*P<*0.05) according to the one-way analysis of variance statistical analysis followed by Newman–Keuls *post hoc* tests after normalization by log transformation (**a**–**k**) or two-way statistical analysis followed by Bonferroni *post hoc* tests (**a**).

We next examined glucose metabolism. Importantly, *IEC MyD88-KO* HFD mice exhibited an improved oral glucose tolerance compared with WT HFD mice (Fig. 4a), an effect that was associated with a decrease in insulin resistance index (Fig. 4b). Impaired glucose metabolism and elevated insulin resistance are associated with hepatic steatosis, macrophage-mediated inflammation of adipose tissues, systemic inflammation and increased production of specific adipokines, such as resistin^16^,^17^.

Moreover, *IEC MyD88-KO* HFD mice exhibited a normalization of the mRNA expression of the chemokine *MCP1* (monocyte chemotactic protein-1) in adipose tissue as well as a significantly reduced mRNA expression of the *CD11c* (cluster of differentiation 11c) subpopulation of macrophages in adipose tissue, which is the primary population of increased adipose tissue macrophages in obesity (Fig. 4c,d)^16^, whereas histological analysis revealed that the number of F4/80-positive cells per adipocyte was slightly increased in HFD mice, without significant effect of the IEC Myd88 deletion (WT: 12.6±2, *IEC MyD88-KO*: 15.5±1.6, WT HFD: 18±2.8 and *IEC MyD88-KO* HFD: 21.9±2 (F4/80-positive cells per 100 adipocytes)), suggesting rather a reduction of M1 polarized macrophages (for example, CD11c cells) than an absolute decrease in infiltrated immune cells in *IEC MyD88-KO*.

In accordance with this hypothesis, we found that plasma interleukin (IL) 6 and resistin levels were significantly increased in HFD-fed mice, whereas both parameters were normalized to basal levels in *IEC MyD88-KO* mice (Fig. 4e,f). Consistent with these observations, we found that plasma MCP1 levels were significantly increased in HFD-fed mice, whereas *IEC MyD88-KO* mice fed with a HFD displayed a reduction of 50% of this parameter (Fig. 4g). Studies have linked *FIAF* (fasting-induced adipose factor) expression with glucose metabolism, fat mass development, gut microbiota and inflammation^18^,^19^,^20^. We have previously found that changing the gut microbiota by using fermentable carbohydrates abolished HFD-induced *FIAF* expression in the adipose tissue, a phenomenon associated with a reduced fat mass and low-grade inflammation^20^. Moreover, it has been shown that *FIAF* expression is decreased by insulin in insulin-sensitive adipose tissue, whereas insulin resistance dampened this effect^21^. Here we found that portal plasma levels of FIAF were significantly increased in HFD-fed mice, whereas *IEC MyD88-KO* HFD mice exhibited a reduction of 12%, but did not reach significance (Fig. 4h).

Interestingly, we found that *FIAF* mRNA expression was similar between groups in the colon of mice; however, we found that HFD feeding doubled *FIAF* expression in the jejunum and in the adipose tissue, whereas IEC MyD88 deletion abolished this effect (Fig. 4i). As *FIAF* mRNA expression has been shown to be regulated by PPARγ (peroxisome proliferator-activated receptor-γ), we measured *PPARg* mRNA expression in the colon, jejunum and adipose tissue. However, we did not find any difference of *PPARg* expression between groups (Supplementary Fig. 2h).

Notably, liver histology revealed that hepatic lipid content was significantly increased in WT HFD mice, whereas the total lipid droplets area was normalized in *IEC MyD88-KO* HFD (Fig. 4j). The later observation was also confirmed by quantifying the triglyceride content, which was decreased by approximately 35% in the *IEC MyD88-KO* HFD group compared with the WT HFD group (WT HFD: 100.89±7.63 and *IEC MyD88-KO* HFD: 66.83±5.42; (nmol mg^−1^ of proteins); *P*=0.002 (Student’s *t*-test)).

### IEC MyD88 controls gut barrier and intestinal endocannabinoids (eCBs)

We next explored the possibility that the reduced metabolic inflammation and insulin resistance may be associated with changes in metabolic endotoxemia (increased serum lipopolysaccharides (LPS) levels) and gut barrier disruption^6^,^7^,^22^. Consistent with the phenotype observed, we found that IEC MyD88 deletion protects mice against HFD-induced metabolic endotoxemia (Fig. 4k), thereby supporting the hypothesis that the deletion improves metabolic inflammation.

**Figure 5:**
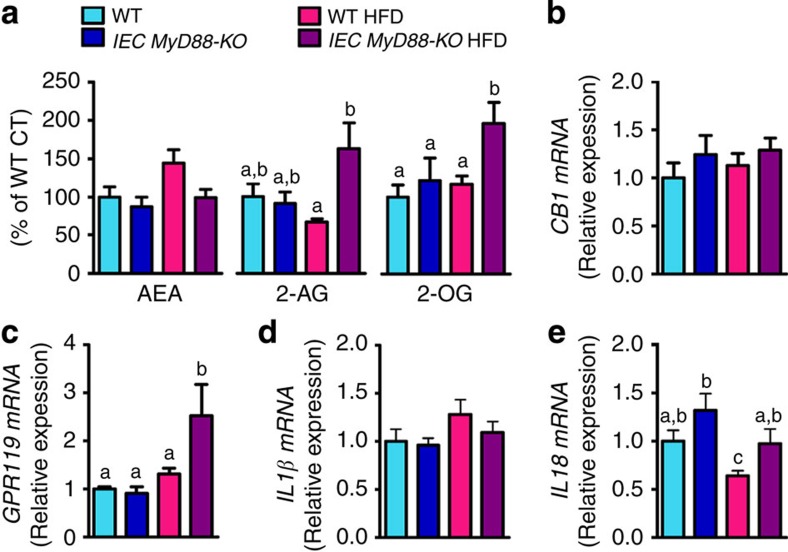
*IEC MyD88* deletion modulates intestinal endocannabinoid system in HFD-fed mice. (**a**) Ileum AEA levels (*n*=10), 2-AG levels (*n*=10) and 2-OG levels (*n*=10). (**b**) *CB1* mRNA in the ileum (*n*=10). (**c**) *GPR119* mRNA in the ileum (*n*=10). (**d**) *IL1β* mRNA in the colon (*n*=10). (**e**) *IL18* mRNA in the jejunum (*n*=15). These data correspond to the results of one experiment. All the replicates represent biological replicates. Data are shown as the means±s.e.m. Data with different superscript letters are significantly different (*P<*0.05) according to the one-way analysis of variance statistical analysis followed by Newman–Keuls *post hoc* tests after normalization by log transformation (data **a**–**e**).

To further elucidate the mechanisms involved in the protection of IEC MyD88 deletion against gut permeability and inflammation, we analysed the intestinal eCB system. We recently identified several links between the eCB system, the gut microbiota and gut barrier function^22^,^23^,^24^. The eCB system is composed of bioactive lipids, with the two main lipids being *N-*arachidonoylethanolamine (anandamide (AEA)) and 2-arachidonoylglycerol (2-AG). We observed that IEC MyD88 deletion abolished the HFD-increased intestinal AEA levels (*P*=0.05 (Student’s *t*-test); Fig. 5a) without affecting the expression of the intestinal CB_1_ receptor (cannabinoid receptor 1; Fig. 5b). We also discovered that intestinal 2-AG levels were doubled in *IEC MyD88-KO* HFD mice compared with WT HFD-fed mice (Fig. 5a). Strikingly, we found that both 2-oleoylglycerol (2-OG) levels and *GPR119* mRNA expression were doubled in the intestine of *IEC MyD88-KO* HFD mice compared with WT HFD-fed mice (Fig. 5a,c). We also measured zonula occludens 1 (encoded by *Tjp1*), *occludin* and *claudin 2* mRNA expression in the gut. We found that occludin expression was similar between groups, whereas *Tjp1* was increased by 25% in HFD-treated mice and normalized in IEC MyD88-deleted mice. *Claudin 2* was decreased upon HFD without any impact of the genetic deletion (Supplementary Fig. 2a–c).

MyD88 is also involved in the signalling of IL1 family members such as IL1β^25^,^26^,^27^ and IL18 (refs 28, 29) and both have been associated with gut barrier function. Therefore, we investigated whether IEC MyD88 deletion affects the expression of *IL1β* and *IL18* in the intestine. We found that IL1β expression was increased by about 30% following HFD feeding, although without reaching statistical significance (Fig. 5d). Conversely, we found that *IL18* expression was significantly decreased following HFD feeding. In both cases, expression changes were normalized by IEC MyD88 deletion (Fig. 5e).

### Modulation of intestinal antimicrobial peptide expression

**Figure 6:**
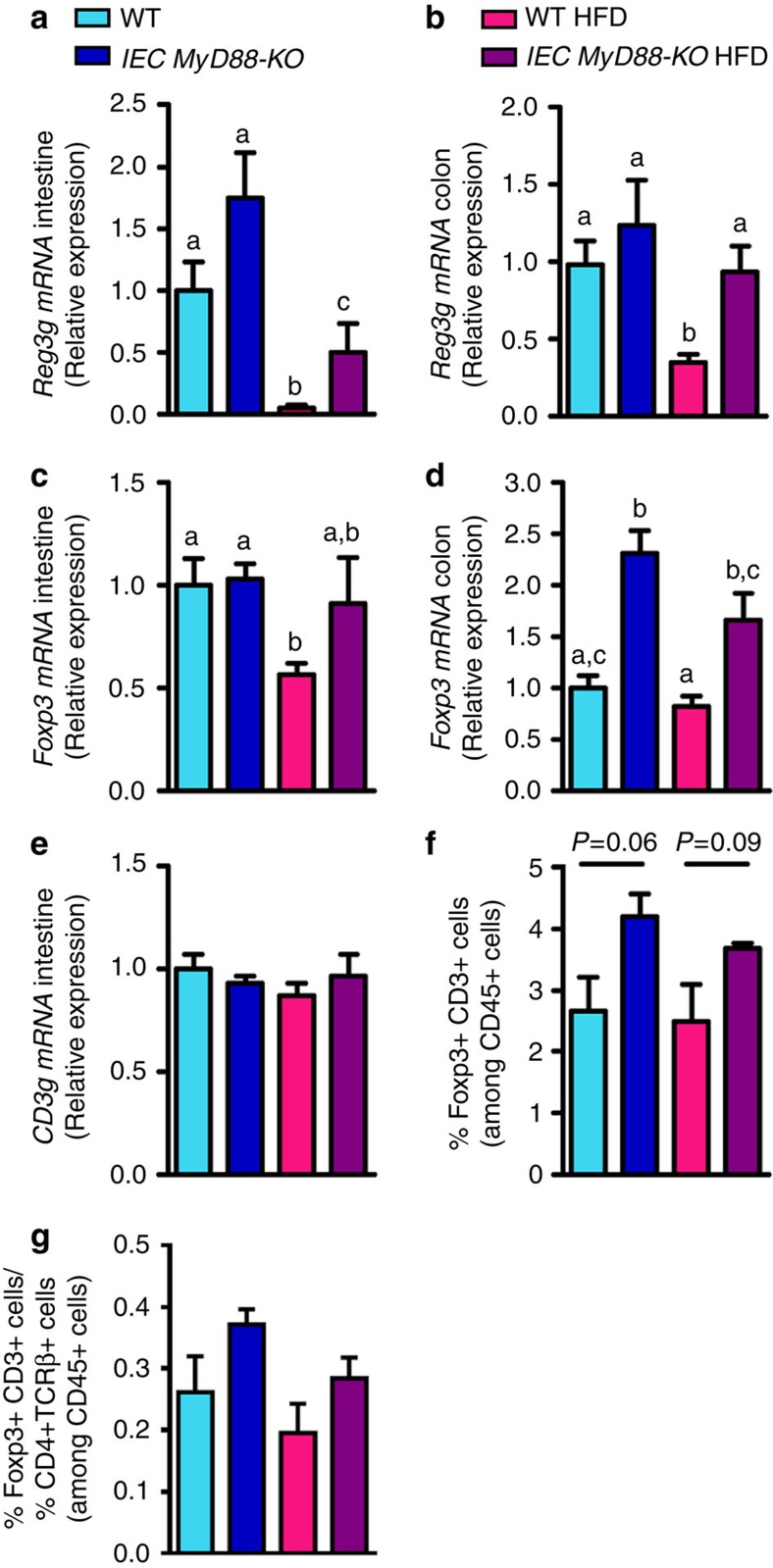
IEC MyD88 deletion regulates markers of intestinal immune system. (**a**) *Reg3g* mRNA in the small intestine (*n*=10). (**b**) *Reg3g* mRNA in the colon (*n*=25). (**c**,**d**) *Foxp3* mRNA in the small intestine and in the colon, respectively (*n*=10). (**e**) *CD3g* mRNA in the small intestine (*n*=10). (**f**) Foxp3^+^ and CD3^+^ cells in the small intestine expressed as a percentage of CD45^+^ cells and measured by flow cytometry (*n*=4). (**g**) Ratio of Foxp3^+^ CD3^+^ cells and TCRβ^+^ cells in the small intestine (*n*=4). Data from **b** correspond to the results of three independent experiments. Data from **a** and **c**–**g** correspond to the results of one experiment. All the replicates represent biological replicates. Data are shown as the means±s.e.m. Data with different superscript letters are significantly different (*P<*0.05) according to the one-way analysis of variance statistical analysis followed by Newman–Keuls *post hoc* tests after normalization by log transformation (data **a**–**e** and **g**). Differences between two groups were assessed using the unpaired two-tailed Student’s *t*-test (data **f**), *P*-value is indicated for each comparison.

Because intestinal MyD88 has been shown to be involved in host-microbial segregation through a mechanism that is dependent on RegIIIγ (regenerating islet-derived 3 γ, encoded by *Reg3g*) antimicrobial protein production^30^,^31^, we investigated the expression of this protein in our experiment. We found that *Reg3g* mRNA expression was decreased in the intestine by the HFD, with a 20-fold decrease and a three-fold decrease in the jejunum and colon, respectively (Fig. 6a,b), thereby confirming our previous findings^22^,^32^. We observed that *Reg3g* mRNA expression was increased by about 75% in the small intestine and 24% in the colon in *IEC MyD88-KO* mice fed a CT, but this increase did not reach significance (Fig. 6a,b). However, we observed an increase in *Reg3g* mRNA expression in the small intestine (10-fold) and in the colon (2.5-fold) of *IEC MyD88-KO* HFD mice compared with WT HFD (Fig. 6a,b). Therefore, this protein may also contribute to the improvement of gut barrier function in *IEC MyD88-KO* HFD mice because it has been suggested that RegIIIγ is involved in the spatial segregation of microbiota and host in the intestine^30^,^31^. Importantly, the production of other antimicrobial proteins was less affected by the intestinal MyD88 deletion (Supplementary Fig. 2d–g).

### IEC MyD88 influences abundance of intestinal regulatory T (Treg) cells

Homeostasis between intestinal gut microbiota and the host is not only achieved by developing immune responses against pathogens such as production of antimicrobial peptides, but also by inducing an immune tolerance to avoid excessive pro-inflammatory responses to commensal bacteria^33^. Indeed, pro-inflammatory responses are suppressed by the action of various subsets of Tregs^34^. The transcription factor that is specific for Tregs and defines this cell lineage is Foxp3 (Forkhead Box transcription factor)^35^. Thus, we measured intestinal *Foxp3* expression to determine if intestinal epithelial MyD88 influences immune tolerance in the intestine. It is well established that obesity is associated with a decrease in Tregs in adipose tissue contributing to inflammation and insulin resistance^36^. In this study, we discovered that HFD-fed obese and diabetic mice also exhibit alteration of Tregs in the intestine as shown by the decreased *Foxp3* mRNA expression in the jejunum (Fig. 6c). Interestingly, the intestinal MyD88 deletion increased intestinal *Foxp3* mRNA expression in HFD-induced obese mice (Fig. 6c). In the colon, we found that *Foxp3* mRNA expression was significantly increased in both *IEC MyD88-KO* CT and *IEC MyD88-KO* HFD mice (Fig. 6d). In order to delineate whether the changes in *Foxp3* mRNA expression were restricted to Treg cells, we measured *CD3g* mRNA expression (a marker of T cells) in the small intestine. We found that *CD3g* mRNA levels were similar between groups (Fig. 6e). Similar to the results observed at the mRNA levels in small intestine and in the colon, after analysing cell populations by using cell sorting, we found that IEC MyD88 deletion increased the number of Foxp3^+^ CD3^+^ cells in the small intestine (Fig. 6f), whereas HFD did not affect this parameter (Fig. 6f). To determine whether the modulation of *Foxp3* mRNA was associated with changes in specific T cells in the intestine, we quantified the ratio of Foxp3^+^ CD3^+^ and TCRβ^+^ cells (two subsets of T cells) in the small intestine^37^ and found a trend similar to the one observed for the Foxp3^+^ CD3^+^ cells (Fig. 6g). Therefore, it is possible that the increased abundance of Treg cells contributes to a better immune response and also participates in the improved metabolism observed in *IEC MyD88-KO* HFD mice. These potent mediators of host defence and barrier maintenance are known to play a role in several hallmarks of adaptive immunity in the intestine^38^.

### Myeloid cell MyD88 deletion does not protect against obesity

**Figure 7:**
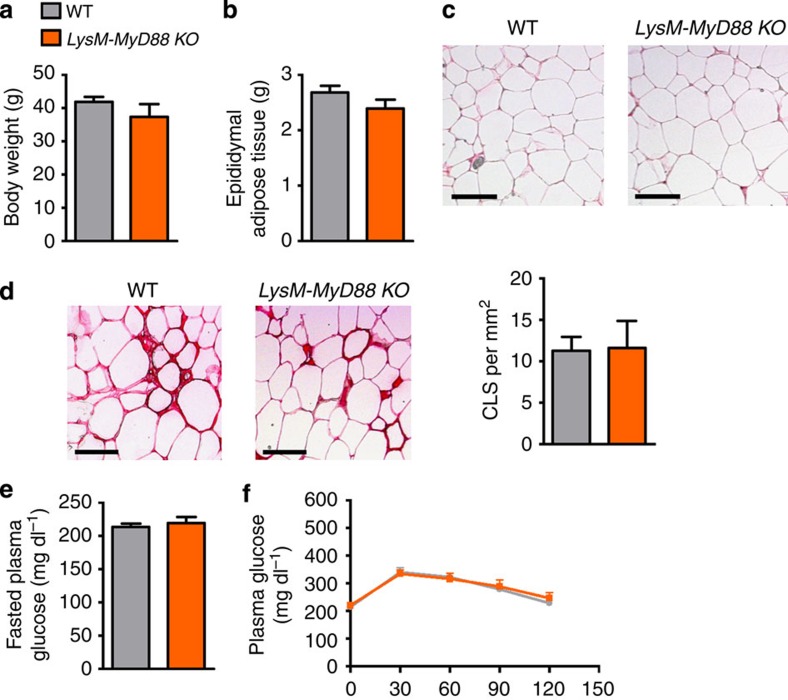
MyD88 deletion in immune cells does not protect against HFD-induced obesity. (**a**) Body weight (g; *n*=4). (**b**) Epididymal fat depot weight (g; *n*=4). (**c**) Representative histology of adipocyte cell size, scale bar, 100 μm (*n*=4). (**d**) Representative histology of crown-like structure (CLS) and quantification of the number of crown-like structures per mm^2^, scale bar, 100 μm (*n*=4). (**e**) Fasted plasma glucose levels (mg dl^−1^; *n*=4). (**f**) Plasma glucose (mg dl^−1^) profile after 1 g per kg intraperitoneal glucose challenge in freely moving mice (*n*=4). These data correspond to the results of one experiment. All the replicates represent biological replicates. Data are shown as the means±s.e.m.

We may not exclude the possibility that the protection observed in *IEC MyD88-KO* mice is not restricted to the epithelial cells but also results from an increased stimulation of MyD88 in intestinal immune cells. Therefore, to assess whether the MyD88 deletion in immune cells contributes to the phenotype, we generated conditional KO depletion of MyD88 in myeloid cell lineages, including monocytes, mature macrophages and granulocytes, by crossing *LysM-Cre* mice with *MyD88-floxed* mice (*LysM-MyD88 KO*). We found that these mice exhibited a similar phenotype compared with WT mice. MyD88 deletion in myeloid cells did not affect HFD-induced body weight gain and fat mass development (Fig. 7a,b). By using histological analysis, we quantified adipocyte size and crown-like structures in adipose tissue. These parameters were not affected by the MyD88 deletion in myeloid cells (Fig. 7c,d). In accordance with these observations, both fasted plasma glucose and glucose tolerance were similar between groups (Fig. 7e,f). Thus, this set of experiments unequivocally showed that intestinal epithelial MyD88 is a primary sensor of the HFD-induced stresses that are able to induce various metabolic disorders.

### Gut microbes from *IEC MyD88-KO* mice protect against obesity

**Figure 8:**
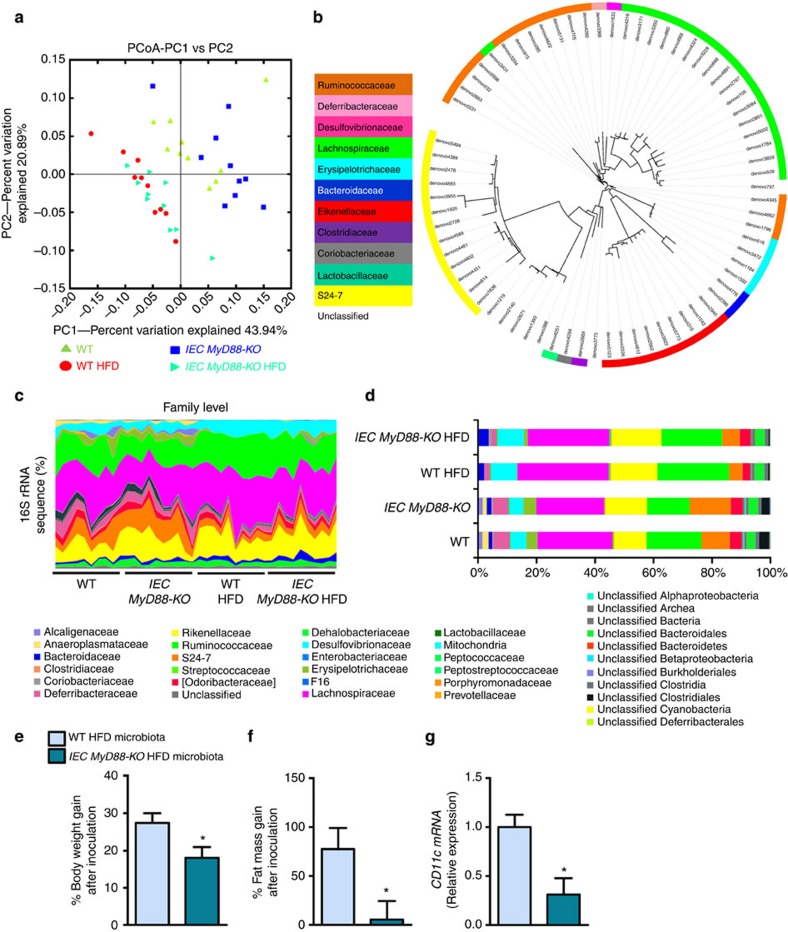
Intestinal MyD88 deletion affects gut bacterial community. Gut bacterial community is analysed by 16S rRNA gene high-throughput sequencing. *IEC MyD88-KO* HFD gut microbiota transfers the partial protection against the obesity phenotype to germ-free mice. (**a**) Principal coordinate analysis based on the weighted UniFrac analysis on operational taxonomic units (OTUs; *n*=10). Each symbol representing a single sample is coloured according to the group. (**b**) OTUs significantly affected by intestinal epithelial MyD88 deletion under HFD. A representative 16S rRNA gene from each of the 72 differentially expressed OTUs in WT HFD versus *IEC MyD88-KO* HFD mice was aligned and used to infer the phylogenetic tree shown in this figure (*n*=10). The colour in front of the OTU indicates the family of the OTU. (**c**) Relative abundances (percentage of 16S rRNA gene sequences) of the different bacterial families in each sample among the WT, *IEC MyD88-KO*, WT HFD and *IEC MyD88-KO* HFD mice (*n*=10). (**d**) Percentage of each indicated family (*n*=10). The different families are represented by different colour codes. **e**–**g** are the results of gut microbiota transfer from WT HFD or *IEC MyD88-KO* HFD mice to WT germ-free mice fed a HFD. (**e**) Percentage of body weight gain after gut microbiota transfer to germ-free mice (%; *n*=5 CT and 4 HFD). (**f**) Percentage of fat mass gain after gut microbiota transfer to germ-free mice (%; *n*=5 CT and 4 HFD). (**g**) *CD11c* mRNA in the adipose tissue (*n*=5 CT and 4 HFD). These data correspond to the results of one experiment. All the replicates represent biological replicates. Data are shown as the means±s.e.m. Data with * are significantly different (*P<*0.05) according to the unpaired two-tailed Student’s *t*-test.

Because changes in serum LPS levels, intestinal eCB tone, intestinal immune cells and intestinal MyD88 deletion have been previously associated with specific gut microbiota composition, we performed a deep gut microbiota analysis using high-throughput sequencing. Consistent with previous studies^6^,^32^,^39^, we found that the HFD diet profoundly affects the gut microbial community (Fig. 8a,c and d; Supplementary Fig. 3a–d and Supplementary Tables 1–3). We also found that 19 bacterial genera were significantly affected by the HFD (Supplementary Table 3) and 11 bacterial genera were significantly affected by the HFD after correction by a false discovery rate (FDR) test according to the Benjamini–Hochberg procedure. We found that IEC MyD88 deletion slightly affected gut microbiota composition under a CT diet (Fig. 8a; and Supplementary Tables 1–3). We did not find major differences on the phylum levels between *IEC MyD88-KO* HFD and WT HFD mice (Supplementary Fig. 3a,b; and Supplementary Table 1). However, we found that the relative abundance of 72 operational taxonomic units (OTUs) was significantly different in *IEC MyD88-KO* HFD mice compared with WT HFD mice (Fig. 8b). IEC MyD88 deletion affected six families (Fig. 8c,d; and Supplementary Table 2) and eight genera compared with WT HFD mice (Supplementary Fig. 3c,d; and Supplementary Table 3) but these differences did not remain significant after FDR test. Among them, the genus *Allobaculum* was decreased in WT HFD mice compared with WT CT mice but was significantly increased in *IEC MyD88-KO* HFD mice compared with WT HFD mice (Supplementary Fig. 3c,d; and Supplementary Table 3). Interestingly, treatment with the plant alkaloid berberine, which prevents obesity and insulin resistance in rats fed a HFD, increased the abundance of *Allobaculum*^40^. Thus, we may not exclude the possibility that IEC MyD88 deletion partially protects against diet-induced obesity through mechanisms associated with metabolic functionalities assumed by gut microbes.

To test this hypothesis, we transferred the gut microbiota from WT HFD or *IEC MyD88-KO* HFD mice into germ-free recipient mice fed a HFD. We found that the gut microbiota from *IEC MyD88-KO* HFD donors reduced diet-induced body weight gain, fat mass development and adipose tissue inflammation compared with the microbiota from WT HFD donors (Fig. 8e,g).

### IEC MyD88 deletion has therapeutic effects against obesity

**Figure 9:**
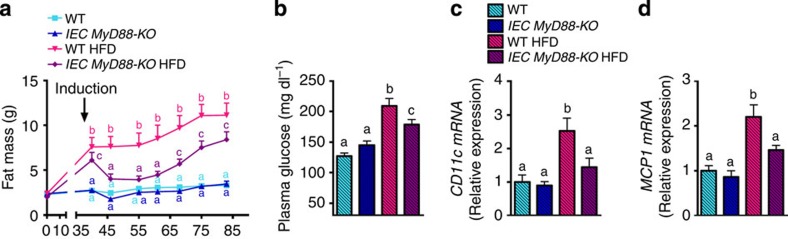
Therapeutic effect of IECMyD88 deletion observed in obese and type 2 diabetic mice. (**a**) Fat mass weight evolution (g) after 6 weeks of dietary treatment and following 7 weeks after intestinal MyD88 deletion (*n*=10). Arrow with the word ‘induction’ indicates tamoxifen injection and thereby intestinal MyD88 deletion. (**b**) Fasted plasma glucose at the end of the experiment (*n*=10). (**c**,**d**) *CD11c* mRNA and *MCP1* mRNA, respectively (*n*=10 per group). These data correspond to the results of one experiment. All the replicates represent biological replicates. Data are shown as the means±s.e.m. Data with different superscript letters are significantly different (*P<*0.05) according to the one-way analysis of variance statistical analysis followed by Newman–Keuls *post hoc* tests after normalization by log transformation (**b**–**d**) or two-way statistical analysis followed by Bonferroni *post hoc* tests (**a**).

Finally, we investigated whether targeting intestinal epithelial MyD88 in diet-induced obese mice might be therapeutically relevant. We found that the induction of IEC-MyD88 deletion after the onset of obesity rapidly and strongly reduced fat mass. Although mice were fed with a HFD for a total of 13 weeks, including 7 weeks after the deletion, we observed that fat mass remained significantly lower in *IEC MyD88-KO* mice and lasted until the end of the experimental period. Importantly, we also found a significantly reduced fasted glycemia and adipose tissue inflammation (Fig. 9a–d).

## Discussion

To date, there is no clear evidence of a unique coupling system between gut microbial-host signals and the onset or the progression of metabolic alterations associated with high-fat feeding.

Here we discovered that intestinal innate immune system and more specifically intestinal epithelial MyD88 is a primary sensor involved in the cross-talks between nutrients, gut microbes and host during diet-induced obesity. We reported that inducible intestinal epithelial cell-specific deletion of MyD88 partially protects against diet-induced fat storage, inflammation and diabetes via mechanisms directly involving the gut microbiota.

During HFD-feeding *IEC MyD88-KO* mice exhibited a lower body weight, fat mass gain and hepatic steatosis, despite similar energy intake and energy absorption. Our data show that this phenotype is explained by an increased energy expenditure observed during HFD feeding; thereby clearly highlighting a novel function of intestinal MyD88 on host energy homeostasis.

We and others have previously shown that HFD feeding increases FIAF expression in the intestine^41^,^42^ and in the adipose tissue^43^. Here we found that IEC MyD88 deletion normalized FIAF expression in the jejunum and in the adipose tissue during HFD feeding. It is worth noting that the reduction of FIAF expression is associated with increased insulin sensitivity, a lower fat mass and reduced inflammation in HFD IEC MyD88 deleted mice. PPARγ has been shown to regulate *FIAF* mRNA expression, here we found that *PPARg* mRNA expression was similar between groups in the colon, jejunum and adipose tissue (Supplementary Fig. 2h), thereby suggesting the implication of another putative mechanism.

Importantly, it has been shown that different TLR’s ligands including fatty acids and LPS stimulate FIAF expression^43^,^44^,^45^. Therefore, we speculate that in absence of MyD88 in the intestinal epithelium both HFD and LPS may not affect the expression of this key factor in the intestine and in the adipose tissue. However, whether intestinal, adipose tissue or circulating FIAF contributes to the phenotype observed in IEC MyD88-deleted mice warrants further investigations.

In addition, we connected the intestinal epithelial innate immune system with the production of anti-inflammatory eCBs (that is, 2-AG and 2-OG). We have previously shown that AEA increases gut permeability in obese mice through a CB_1_-dependent mechanism^23^ and that 2-AG reduces metabolic endotoxemia and systemic inflammation in a colitis model^46^. Our data show that AEA was decreased in the absence of IEC MyD88, whereas 2-AG and 2-OG were increased specifically during HFD feeding. Importantly, 2-OG is another lipid that is related to the eCB system and binds to the GPR119 receptor, thereby stimulating the release of gut peptides such as glucagon-like peptide-1 and glucagon-like peptide-2 released from intestinal L-cells^47^,^48^, respectively, involved in glucose homeostasis and gut barrier function. This suggests that the improved glucose homeostasis, the reduced metabolic endotoxemia and low-grade inflammatory tone observed in *IEC MyD88-KO* mice are associated with the regulation of intestinal eCB system.

The expression of the tight junction markers measured in the present study was not significantly affected by IEC MyD88 deletion. However, it has been proposed that intestinal epithelial cells produce IL18 in order to contribute to the preservation of the intestinal barrier, mainly by inducing proliferation and thus enhancing the regeneration of the damaged epithelium^28^,^29^. Interestingly, we found that HFD feeding decreases IL18 expression in the intestine, whereas IEC MyD88 deletion normalized this parameter. Thus, these data suggest that intestinal MyD88 contributes to regulate *IL18* expression during HFD feeding and thereby contributes to improve gut barrier function.

We found that IEC MyD88 deletion reduces body weight gain and fat mass development despite similar energy intake and energy absorption. Conversely, Kleinridders *et al.* found that a deletion of MyD88 in the central nervous system results in partial protection against obesity^14^. The authors suggested that the mechanism of protection against diet-induced obesity is driven by changes in food intake and not by a mechanism involving energy expenditure^14^. Thus, these data suggest that the impact of MyD88 deletion on energy intake and energy expenditure may be considered as tissue dependent. Therefore, these data let us postulating that in the brain fatty acids signalling through MyD88 controls leptin sensitivity and appetite, whereas in the intestine MyD88 controls energy metabolism via several mechanisms including cross-talks with gut microbes.

We found that IEC MyD88 deletion induces changes in the gut microbial community. It has been previously observed that mice lacking whole-body MyD88 have an altered microbial composition in the gut^10^,^12^. Antimicrobial peptide production has been associated with innate immunity and gut microbes^32^,^49^,^50^,^51^. These immune factors play a major role in host–gut microbiota interactions. Indeed, through these immune factors, the host controls its interactions with the gut microbiota and thereby shapes its microbial communities^50^. Importantly, we confirmed that HFD feeding decreases intestinal *Reg3g* expression^22^. However, *IEC MyD88-KO* mice were protected against diet-induced downregulation of *Reg3g* expression, which is not consistent with previous studies^30^,^31^. We postulate that this major difference occurs mainly because our mouse model is inducible and, therefore, the MyD88 deletion is induced in adult mice, thereby allowing normal development of the immune system and avoiding any adaptation of the immune system and the gut microbiota during the development of the mice. We also found that IEC MyD88 deletion increased the number of Foxp3^+^ CD3^+^ cells in the intestine under normal and high-fat conditions. Thus, we postulate that the increased abundance of Treg cells contributes to improve gut barrier function in *IEC MyD88-KO* mice.

We show that IEC MyD88 deletion changes the gut microbiota. More importantly, we demonstrated that transplanting gut microbes from *IEC MyD88-KO* mice into germ-free mice replicated the protection against diet-induced metabolic disorders, unequivocally showing that host intestinal immune system (that is, IEC MyD88) controls the gut microbiota, which in turn affects host metabolism. In future studies, it will be crucial to examine whether one or several specific microbes or metabolites are involved in such effects. Finally, we show that targeting IEC MyD88 in diet-induced obese and type 2 diabetic animals strongly reduces fat mass, improves glucose metabolism and reduces inflammation. However, we found that IEC MyD88-deleted mice progressively regained fat mass, although the total fat mass remained significantly lower 8 weeks after MyD88 deletion. We may not rule out that compensatory mechanisms may have occurred by changing downstream markers of the MyD88-dependent and -independent pathways. Notably, *TRAF* mRNA expression was not affected by IEC MyD88 deletion (Supplementary Fig. 4), thus, this observation deserves further investigations.

Here we provide a novel pathway for diet-induced obesity, type 2 diabetes and metabolic disorders and pointed out the major role played by MyD88 in the intestinal epithelial cells on gut microbiota modulation and host energy metabolism.

Importantly, we may speculate that this phenomenon could also occur in humans. Therefore, in a clinical point of view, future therapeutic approaches based on gut microbiota modulation or pharmacological targeting of intestinal epithelial MyD88 modulation may be of utmost interest. So far, there are no human studies showing that host intestinal epithelial MyD88 controls gut microbiota composition and that this is associated with metabolic disorders. Thus, whether such alterations in the IEC MyD88 pathway occur in human intestine and thereby contribute to the onset of obesity and related disorders via gut microbiota-dependent mechanisms requires further investigations.

In conclusion, we discovered a novel function of intestinal epithelial MyD88. We show that targeting intestinal epithelial MyD88 confers protection or therapeutic effects against diet-induced metabolic disorders. Thus, intestinal epithelial MyD88 acts as a metabolic sensor that switches host metabolism during diet-induced obesity via mechanisms involving the gut microbiota. These unique features render intestinal epithelial MyD88, an attractive target for preventing or treating diet-induced obesity and metabolic disorders.

## Methods

### Mice

*Generation of IEC MyD88-KO*. The inducible intestinal epithelial MyD88-deleted mice (IEC MyD88-KO) were generated by crossing mice bearing a tamoxifen-dependent Cre recombinase expressed under the control of the villin promoter (*Villin Cre-ERT2*) with mice harbouring a *loxP*-flanked *Myd88* allele (C57BL/6 background, Jackson-Laboratory). The deletion was induced at 9 weeks of age by intra peritoneal (i.p.) injection of 100 μl tamoxifen (10 mg ml^−1^) for 5 consecutive days. Tamoxifen was prepared by addition of ethanol to 50 mg of tamoxifen (tamoxifen-free base, MP Biomedicals) to obtain a 10 mg per 100 μl of tamoxifen suspension. A 10 mg ml^−1^ tamoxifen solution was prepared by addition of filtered sunflower oil, followed by 30 min sonication. The 10 mg ml^−1^ of tamoxifen solution was stored at 4 °C for up to 1 week. The tamoxifen solution was sonicated just before use^52^.

*IEC MyD 88-KO HFD experiment*. Three sets of 10-week-old WT or *IEC MyD88-KO* male mice (40 mice, *n*=10 per group) were housed in groups of 2 mice per cage (filter-top cages), with free access to food and water. The mice were fed a CT (AIN93Mi; Research diet) or a HFD (60% fat and 20% carbohydrates (kcal per 100 g), D12492, Research diet). Treatment continued for 8 weeks. This experiment has independently been realized three times. Two types of control mice were used in these experiments: WT littermate mice injected with Tamoxifen and mice harbouring the *Villin Cre MyD88* construct injected with the vehicle.

Body weight and body composition were assessed once a week by using 7.5 MHz time domain-NMR (TD-NMR; LF50 minispec, Bruker).

*IEC MyD 88-KO HFD experiment for indirect calorimetry studies*. A fourth set of WT or *IEC MyD88-KO* male mice (12 mice, *n*=6 per group) were housed 1 mouse per cage, with free access to food and water. The mice were fed a CT (AIN93Mi; Research diet). At 10-week old, the mice were placed for 1 week in metabolic chambers (Labmaster, TSE Systems GmbH). For the next 8 weeks, mice were fed a HFD (60% fat and 20% carbohydrates (kcal per 100 g), D12492, (Research diet). At the end of the treatment, mice were again placed for 1 week in metabolic chambers (Labmaster, TSE Systems GmbH). At the end of the experiment, feces energy content has been measured by calorimetric bomb (Mouse Clinical Institute, 67404 Illkirch, France).

*Therapeutic effect of IEC MyD 88-KO induction*. A fourth set of 10-week-old male mice (*n*=40) were fed a CT (*n*=20) or a HFD (*n*=20) for 6 weeks in order to induce obesity, type 2 diabetes and low-grade inflammation. After 6 weeks of treatment, IEC MyD88 deletion was induced in order to study its potential therapeutic effect. Mice were kept under CT or HFD and followed for additional 7 weeks.

*Microbiota transplantation experiments*. Caecal contents from five WT HFD male mice were transplanted into germ-free mice fed with a HFD and caecal contents from five *IEC MyD88-KO* HFD mice were transplanted into germ-free mice fed with HFD. Caecal content donors were 10-week-old WT HFD or *IEC MyD88-KO* mice fed a HFD for 8 weeks as described in *IEC MyD 88-KO HFD* experiment.

Each caecal content (150 mg) was sampled in an anaerobic chamber and suspended in PBS (2.5 ml per caecum). The gut microbes were then administered (0.15 ml per mouse) immediately to germ-free mice (7-week-old Swiss-Webster male) from Taconic. Transplanted mice were maintained in individualized ventilated cages (IVC AERO GM500, Tecnilab-BMI) and fed with a HFD (60% fat and 20% carbohydrates (kcal per 100 g), D12492, Research diet) for 3 weeks.

Body weight and body composition were assessed once a week by using 7.5 MHz TD-NMR (LF50 minispec, Bruker).

*LysM-Cre and MyD88 floxed mice*. Male *LysM-Cre* transgenic mice were bred with mice harbouring a *loxP*-flanked *Myd88* allele (C57BL/6 background, Jackson-Laboratory). 12-week-old mice were fed HFD (45 kcal% fat, mostly lard; D10011202; Research Diets) for 12 weeks with free access to food and water. Floxed MyD88 mice not carrying the *LysM-Cre* allele were used as negative controls.

### Ethical approval

All mice experiments were approved by and performed in accordance with the guidelines of the local ethic committee (ethic committee of the Université Catholique de Louvain for animal experiments specifically approved this study that received the agreement number 2010/UCL/MD/022 and 2014/UCL/MD/010). Housing conditions were specified by the Belgian Law of 29 May 2013 regarding the protection of laboratory animals (agreement number LA1230314).

### Tissue sampling

The animals have been anaesthetized with isoflurane (Forene, Abbott) before exsanguination and tissue sampling, then mice were killed by cervical dislocation. Adipose depots (epididymal, subcutaneous and mesenteric) and liver were precisely dissected and weighed. The portal serum, the cava vein plasma, the intestinal segments (jejunum, ileum and colon), caecal content, liver and adipose tissues were immersed in liquid nitrogen, and stored at −80 °C, for further analysis.

### Indirect calorimetry studies

Mice were analysed for whole energy expenditure, oxygen consumption and carbon dioxide production, RQ (vCO_2_/vO_2_), food intake and spontaneous locomotor activity (counts per h) using calorimetric (Labmaster, TSE Systems GmbH). Activity was recorded using infrared light beam-based locomotion monitoring system. Mice were individually housed and acclimated to the chambers for 48 h before experimental measurements.

Data analysis was performed using O_2_ consumption (ml per h), CO_2_ production (ml per h) and energy expenditure (kcal per h) subsequently expressed as a function of whole lean body mass^53^. Lean body mass was acquired using an Echo Medical systems EchoMRI 100 (Whole Body Composition Analyser, EchoMRI)^54^.

### RNA preparation and real-time qPCR analysis

Total RNA was prepared from tissues using TriPure reagent (Roche). Quantification and integrity analysis of total RNA were performed by analysing 1 μl of each sample in an Agilent 2100 Bioanalyzer (Agilent RNA 6000 Nano Kit, Agilent). cDNA was prepared by reverse transcription of 1 μg total RNA using a Reverse Transcription System kit (Promega). Real-time PCR was performed with the StepOnePlus real-time PCR system and software (Applied Biosystems) using Mesa Fast qPCR (Eurogentec) for detection according to the manufacturer’s instructions. RPL19 RNA was chosen as the housekeeping gene. All samples were performed in duplicate in a single 96-well reaction plate, and data were analysed according to the 2^−ΔΔCT^ method. The identity and purity of the amplified product were assessed by melting curve analysis at the end of amplification. The primer sequences for the targeted mouse genes are presented in Supplementary Table 4.

### DNA isolation from mouse caecal samples and sequencing

Metagenomic DNA was extracted from the caecal content using a QIAamp-DNA stool mini-kit (Qiagen) according to the manufacturer’s instructions. The V1-V3 region of the bacterial 16S rRNA gene was amplified using barcoded primers 27f (5′-CCTATCCCCTGTGTGCCTTGGCAGTCTCAG-3′) and 534r (5′-ATTACCGCGGCTGCTGG-3′)^55^ and high-throughput sequencing of purified amplicons were analysed on a Roche FLX Genome Sequencer using the Titanium chemistry (DNAVision). Resulting reads were processed through QIIME v1.7.0 pipeline^56^. OTUs were identified using the uclust consensus taxonomy classifier with a 0.97 threshold against the Greengenes database (Fig. 8) or using mother (Supplementary Tables 1–3). Principal coordinate analysis was calculated using weighted UniFrac distance. Phylogenetic tree was generated using QIIME 1.7.0 and visualized using iTOL v2.2.2 (ref. 57).

### Glucose tolerance test

Oral glucose tolerance tests were performed after 6 weeks. Food was removed 2 h after the onset of the daylight cycle and mice were treated after a 6-h fasting period with an oral gavage glucose load (2 g per kg body weight) and blood glucose levels were measured before oral glucose load and 15, 30, 60, 90 and 120 min after oral glucose load. Blood glucose was determined with a glucose metre (Accu Check Aviva, Roche) on blood samples collected from the tip of the tail vein^6^. For *LysM-Cre* mice, glucose tolerance tests were performed by injecting glucose (1 g per kg body weight) intraperitoneally after a 4-h fast. Tail blood samples were collected at 0, 30, 60, 90 and 120 min and blood glucose levels were determined using a HemoCue glucose 201+ analyser (HemoCue).

### Insulin resistance index

Plasma insulin concentration was determined in 5 μl of plasma using an ELISA kit (Mercodia) according to the manufacturer’s instructions. Insulin resistance index was determined by multiplying the area under the curve of both blood glucose (0 and 120 min) and plasma insulin (0 and 15 min) obtained following the oral glucose tolerance test.

### Biochemical analyses

Portal vein blood LPS concentration was measured by using Endosafe-MCS (Charles River Laboratories) based on the limulus amaebocyte lysate kinetic chromogenic methodology that measures colour intensity directly related to the endotoxin concentration in a sample. Serum was diluted one-tenth with endotoxin-free buffer to minimize interferences in the reaction (inhibition or enhancement) and heated 15 min at 70 °C. Each sample was diluted 1/70 or 1/100 with endotoxin-free limulus amaebocyte lysate reagent water (Charles River Laboratories) and treated in duplicate and two spikes for each sample were included in the determination. All samples have been validated for the recovery and the coefficient variation. The lower limit of detection was 0.005 EU ml^−1^.

Plasma adipokines (leptin, resistin, MCP1 and IL6) were determined by using a Bio-Plex Milliplex kit (Millipore) and measured by using Luminex technology (Bio-Rad Bioplex; Bio-Rad) following the manufacturer’s instructions. Portal vein plasma FIAF levels were determined by using ELISA (Proteogenix).

### Fluorescence-activated cell sorting (FACS)

Intestines were dissected and cut longitudinally after removing the Peyer’s patches and the fat. Small pieces of intestine were incubated with EDTA (2.5 mM) and dithiothreitol (72.5 μg ml^−1^) during 20 min at 37 °C with shaking (200 r.p.m.). Samples were strained to eliminate intraepithelial lymphocytes by mechanical disruption. Tissue was further digested with 5 mg ml^−1^ liberase (Roche) and 0.05% DNase I (Roche), at 37 °C for 30 min. Digested tissue was passed through 70-μm cell strainer and lymphocytes were further enriched by centrifugation in a solution of 30% Percoll to eliminate epithelial cells. Then the supernatant was eliminated and the cells were incubated with antibodies for FACS staining.

For FACS staining, 1 million cells were preincubated with 10 mg ml^−1^ purified rat anti-mouse CD16-CD32 mAb (Fc Block) before incubation with specific antibodies for CD45 (30-F11 conjugated with PerCP), CD3 (17A2 conjugated with AlexaFluor700), CD4 (RM4-5 conjugated with PECy7) and for TCRb (H57-597 conjugated with FITC; all from BioLegend) and with a dead cell marker (LIVE/DEAD Fixable Near-IR Dead Cell Stain Kit, Life). The antibodies were added for 1 h at 4 °C at 2 mg ml^−1^.

For the FOXP3 intracellular staining, we used the Mouse Regulatory T Cell Staining Kit from eBioscience.

Cells were gated, based on forward and side scatter and on living cells and CD45^+^ cells with a FACS Fortessa (BD Biosciences). Postacquisition analysis was performed using FlowJo software (Tree Star).

### Histological analysis

Paraffin-embedded epididymal WAT sections (5 μm) were deparaffinized and processed for antigen retrieval with a 2100 Retriever using 13 DIVA solution and Hot Rinse (HistoLab Products AB). Endogenous peroxidase activity was quenched by incubating slides in 0.3% H_2_O_2_ in PBS for 30 min before blocking in 5% rabbit serum, 1% bovine serum albumin and 0.1% Triton X-100 at room temperature for 30 min. In *LysM-MyD88* mice, macrophages were stained with MAC-2/galectin-3 antibody (CL8942AP, Cedarlane Laboratories; diluted 1:500 in blocking buffer overnight at 48 °C, which was detected with a biotinylated anti-rat (10 mg ml^−1^) antibody. Immune complexes were detected by VECTASTAIN Elite ABC reagent followed by colour development using NovaRed substrate solution (Vector Laboratories), according to the manufacturer’s instructions, and counterstained with haematoxylin. Crown-like structures and adipocyte size were counted in 1.5–6 mm^2^ of histological sections per mouse.

Adipocyte size (haematoxylin and eosin staining) and macrophage infiltration (F4/80: ab6640; Abcam) were determined in a blind manner by the investigator and quantified using ImageJ software (National Institutes of Health, version 1.48r). Hepatic lipid content was determined by using Oil red O staining. Five slices per mouse were counted with ten mice per group. Quantification was performed using ImageJ software.

### Measurement of eCBs intestinal levels

Ileum tissues were homogenized in CHCl_3_ (10 ml), and a deuterated standard (200 pmol) was added. Methanol (5 ml) and H_2_O (2.5 ml) were added, and the lipids were then extracted by vigorous mixing. After centrifugation, the organic layer was recovered, dried under a stream of N_2_ and purified by solid-phase extraction using silica, followed by elution with an EtOAc-Acetone (1:1) solution. The resulting lipid fraction was analysed by high-performance liquid chromatography-MS using an LTQ Orbitrap mass spectrometer (ThermoFisher Scientific) coupled to an Accela HPLC system (ThermoFisher Scientific). Analyte separation was achieved by using a C-18 Kinetex C-18 column (5 μm, 4.6 × 150 mm; Phenomenex) and a C18 pre-column. Mobile phases A and B were composed of MeOH/H_2_O/acetic acid 75:25:0.1 (v/v/v) and MeOH/acetic acid 100:0.1 (v/v), respectively. The gradient (0.5 ml min^−1^) was designed as follows: transition from 100% A to 100% B linearly over 15 min, followed by 10 min at 100% B and subsequent re-equilibration at 100% A. We performed mass spectrometry analysis in the positive mode with an APCI ionisation source. The capillary and APCI vaporiser temperatures were set at 250 and 400 °C, respectively. The eCBs were quantified by isotope dilution using their respective deuterated standard (showing identical retention times). The calibration curves were generated as described and the data were normalized by tissue sample weight^23^.

### Statistical analysis

Data are expressed as means±s.e.m. Data were analysed for similarity of variance between groups using the Bartlett test. Differences between two groups were assessed using the unpaired two-tailed Student’s *t*-test. Data sets involving more than two groups were assessed by analysis of variance followed by Newman–Keuls *post hoc* tests after normalization by Log transformation. Data with different superscript letters are significantly different *P<*0.05, according to the *post-hoc* analysis of variance statistical analysis. Data were analysed using GraphPad Prism version 5.00 for windows (GraphPad Software). Data related to the gut microbiota were analysed using JMP 8.0.1 (SAS Institute, Inc) and R 3.0.2 (The R Foundation) with the RStudio 0.97.310 packages. The microbial compositions were statistically significant at *P<*0.05 using *t*-test and data were corrected by a FDR test according to the Benjamini-Hochberg procedure with *α*<0.05. Results were considered statistically significant when *P<*0.05.

## Additional information

**How to cite this article:** Everard, A. *et al.* Intestinal epithelial MyD88 is a sensor switching host metabolism towards obesity according to nutritional status. *Nat. Commun.* 5:5648 doi: 10.1038/ncomms6648 (2014).

## Supplementary Material

Supplementary Information Supplementary Figures 1-4, Supplementary Tables 1-4
