# Supplementary material for: Intestinal epithelial MyD88 is a sensor switching host metabolism towards obesity according to nutritional status
Source: Nat Commun. 2014 Dec 5;5:5648. doi: 10.1038/ncomms6648 (PMC4268705; doi:10.1038/ncomms6648)
Supplement: Supplementary Information — Supplementary Figures 1-4, Supplementary Tables 1-4 [file ncomms6648-s1.pdf]

Supplementary Figures and Tables

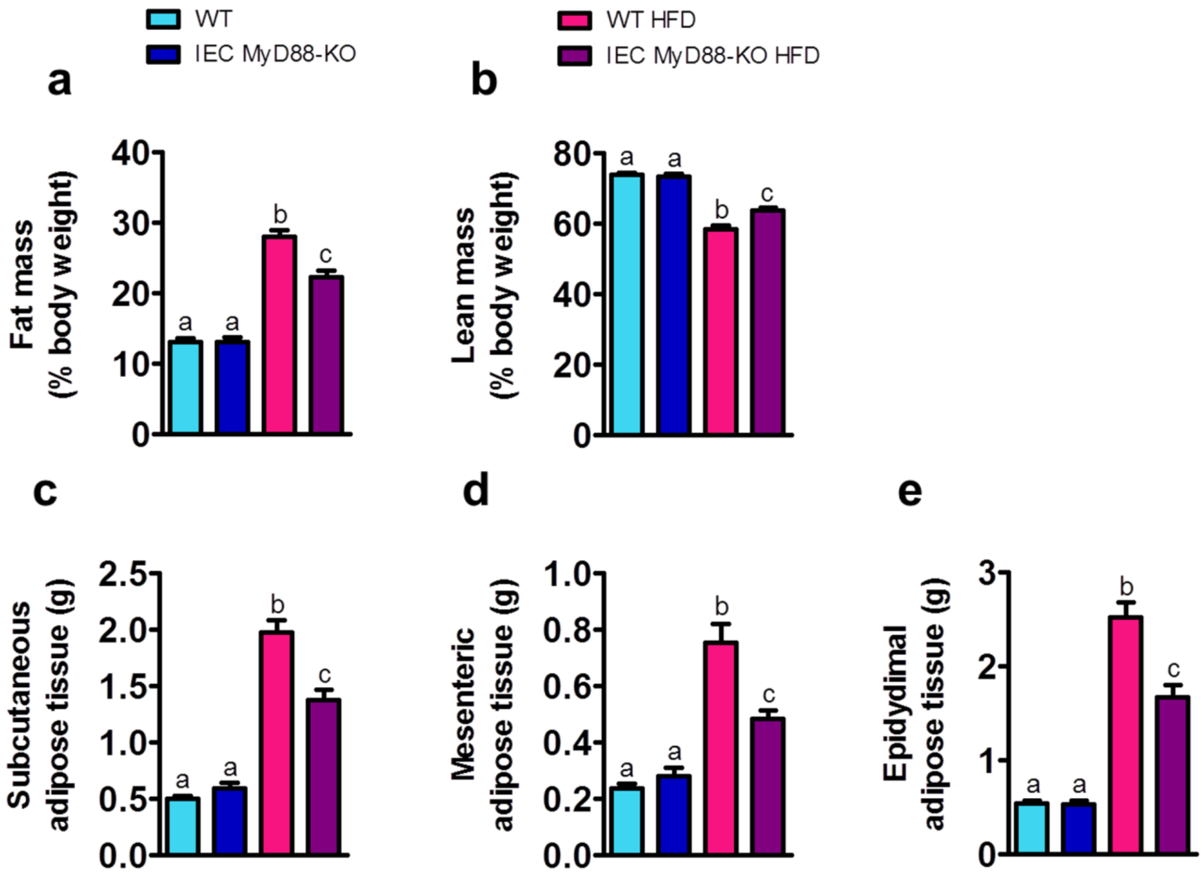

Supplementary Figure 1. Intestinal epithelial MyD88 deletion decreases fat mass under HFD. (**a**) Final fat mass expressed as a percentage of final body weight (n=25). (**b**) Final lean mass expressed as a percentage of the final body weight (n=25). Subcutaneous (**c**), mesenteric (**d**) and epididymal (**e**) fat depot weights (g). These data correspond to the results of three independent experiments. All the replicates represent biological replicates. Data are shown as the means ± s.e.m. Data with different superscript letters are significantly different ($P < 0.05$) according to the ANOVA one-way statistical analysis followed by Newman-Keuls post hoc tests after normalization by Log transformation.

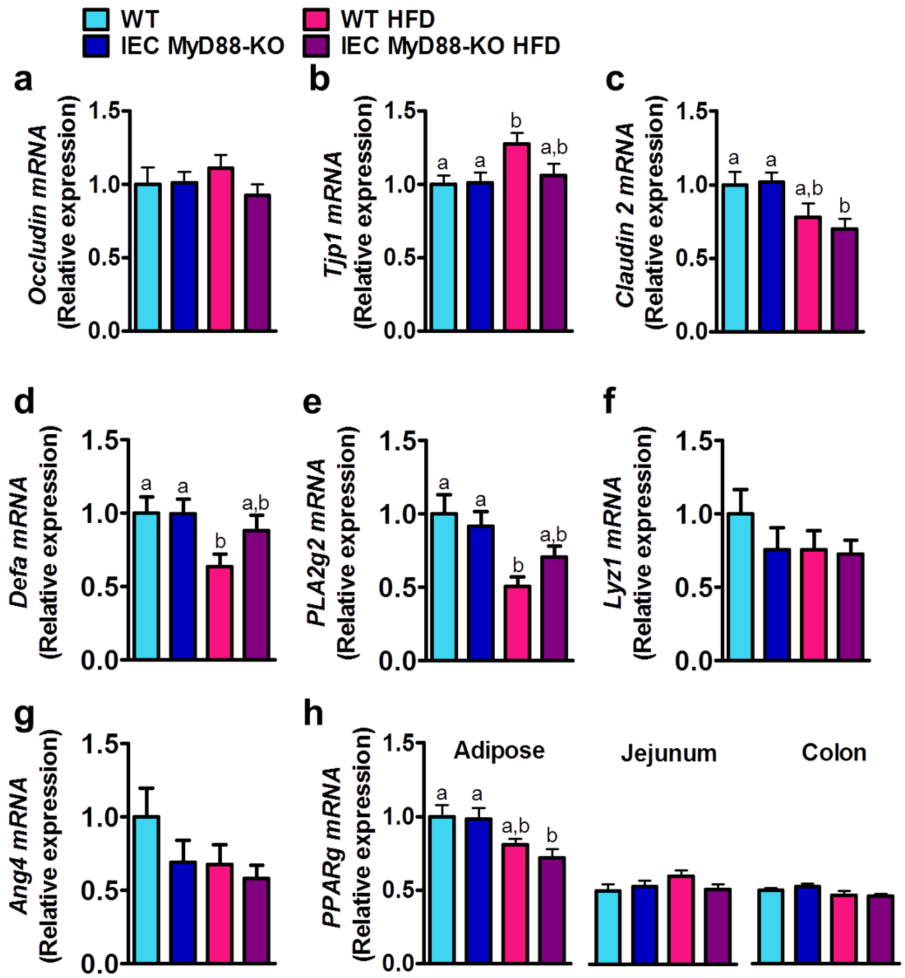

Supplementary Figure 2. **Expression of *Occludin, Tjp-1, Claudin 2, Defa, PLA2g2, Lyz*1, *Ang4,* and *PPARg.*** (**a**) Occludin mRNA expression (encoded by *occludin*) (n=10), (**b**) zonula occludens 1 (*Tjp-1*) mRNA expression (encoded by *ZO-1*) (n=10), (**c**) claudin 2 mRNA expression (encoded by *claudin 2*) (n=10) (**d**) α-defensins mRNA expression (encoded by *Defa*) (n=10), (**e**) Phospholipase A2 group IIA mRNA expression (encoded by *Pla2g2a*) (n=10), (**f**) Lysozyme C mRNA expression (encoded by *Lyz1*) (n=10) and (**g**) Angiogenin 4 mRNA expression (encoded by *Ang4*) (n=10) measured by RT-qPCR in the jejunum of mice. (**h**) Peroxisome proliferator-activated receptor gamma (*PPARg*) measured by RT-qPCR in the adipose tissue, jejunum and colon of mice (n=10). These data correspond to the results of one experiment. All the replicates represent biological replicates. Data are shown as the means ± s.e.m. Data with different superscript letters are significantly different ($P < 0.05$) according to the ANOVA one-way statistical analysis followed by Newman-Keuls post hoc tests after normalization by Log transformation.

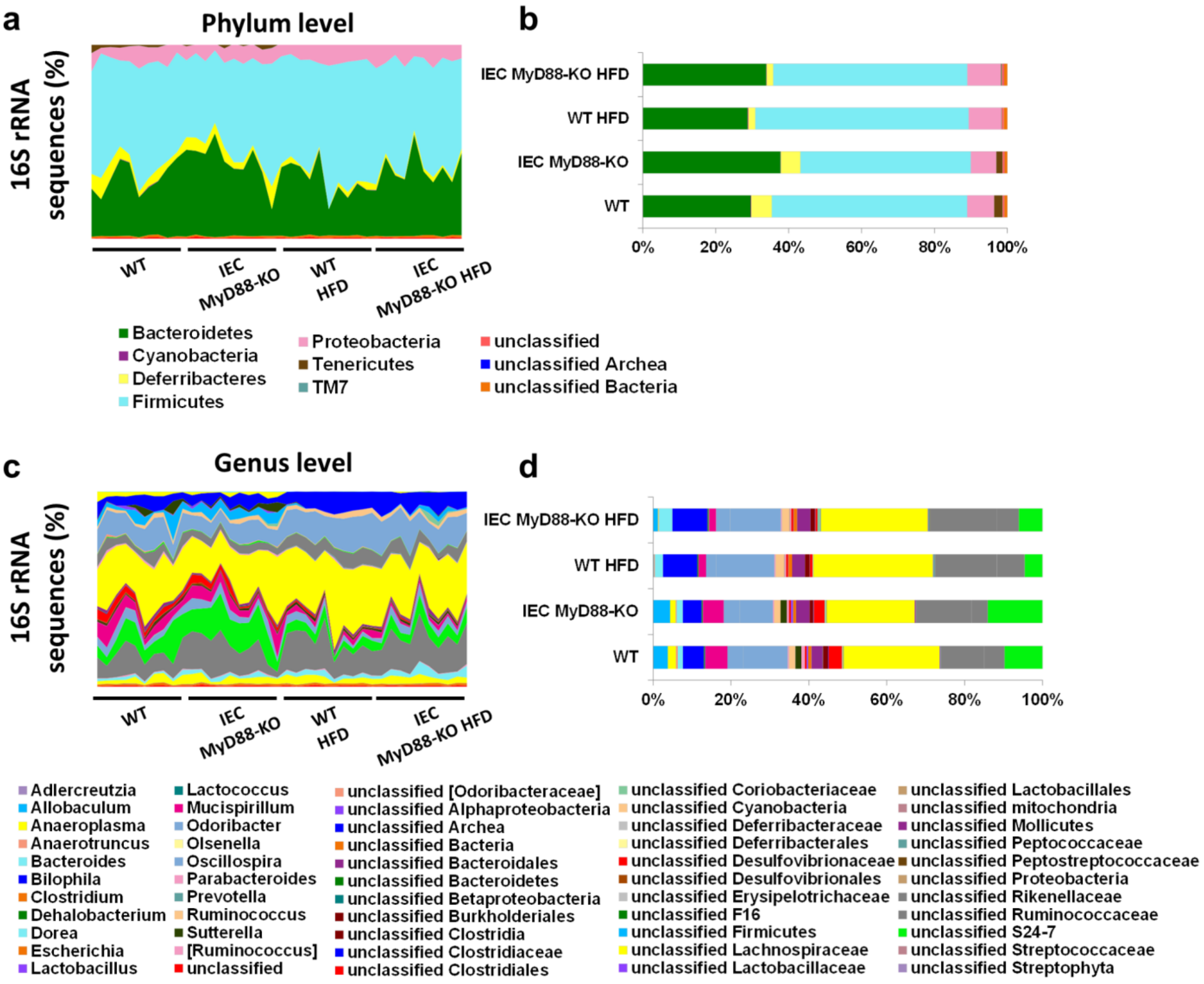

Supplementary Figure 3. Intestinal MyD88 deletion affects gut bacterial community analyzed by 16S rRNA high-throughput sequencing. (**a**) Relative abundances (percentage of 16S rRNA sequences) of the different bacterial phyla in each sample among the WT, IEC MyD88-KO, WT HFD and IEC MyD88-KO HFD mice (n=10). (**b**) Percentage of each community contributed by the indicated phylum (n=10). (**c**) Relative abundances (percentage of 16S rRNA sequences) of the different bacterial genera in each sample among the WT, IEC MyD88-KO, WT HFD and IEC MyD88-KO HFD mice (n=10). (**d**) Percentage of each community contributed by the indicated genus (n=10). In all the figures the different phyla and genera are represented by different color codes. These data correspond to the results of one experiment. All the replicates represent biological replicates.

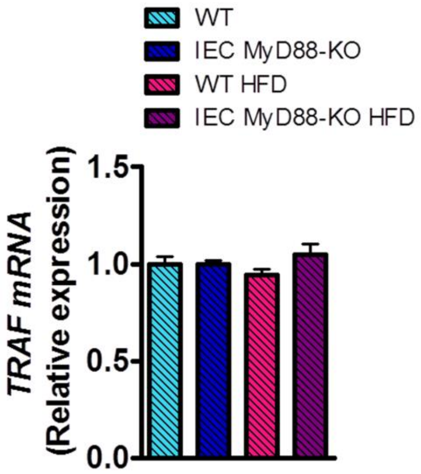

Supplementary Figure 4. Therapeutic effect of intestinal MyD88 deletion observed in obese and type 2 diabetic mice. *TRAF* mRNA measured in the colon at the end of the experiment (n=10). These data correspond to the results of one experiment. All the replicates represent biological replicates. Data are shown as the means ± s.e.m. Data with different superscript letters are significantly different ($P < 0.05$) according to the ANOVA one-way statistical analysis followed by Newman-Keuls post hoc tests after normalization by Log transformation.

Supplementary Table 1: Relative abundance of the bacterial phyla found in the different groups (% of 16S rRNA sequences)

	WT		IEC MyD88-KO			
	Mean	SEM	Mean	SEM	changes (%)	p-value
**Actinobacteria**	0,13	0,08	0,34	0,15	151,89	0,24962
**Bacteroidetes**	29,94	2,51	38,22	3,40	27,65	0,06586
**Cyanobacteria**	0,00	0,00	0,00	0,00	-42,48	0,65350
**Deferribacteres**	5,62	0,85	5,25	0,90	-6,56	0,76811
**Firmicutes**	52,06	2,11	45,27	2,46	-13,04	0,05067
**Proteobacteria**	7,13	0,86	6,79	0,55	-4,80	0,73960
**Tenericutes**	2,15	0,44	1,40	0,34	-34,94	0,19231
**TM7**	0,07	0,01	0,06	0,01	-18,97	0,52643
**Unclassified**	0,50	0,07	0,57	0,05	15,14	0,35580
**unclassified Bacteria**	2,39	0,12	2,10	0,18	-12,27	0,18891

	WT		WT HFD			
	Mean	SEM	Mean	SEM	changes (%)	p-value
**Actinobacteria**	0,13	0,08	0,04	0,01	-71,07	0,22517
**Bacteroidetes**	29,94	2,51	29,25	2,95	-2,31	0,86032
**Cyanobacteria**	0,00	0,00	0,01	0,01	378,28	0,26689
**Deferribacteres**	5,62	0,85	1,94	0,39	-65,41	0,00094
**Firmicutes**	52,06	2,11	57,12	2,61	9,71	0,14947
**Proteobacteria**	7,13	0,86	8,75	0,54	22,75	0,12591
**Tenericutes**	2,15	0,44	0,01	0,01	-99,36	0,00013
**TM7**	0,07	0,01	0,03	0,01	-52,36	0,05468
**Unclassified**	0,50	0,07	0,48	0,04	-4,14	0,79150
**unclassified Bacteria**	2,39	0,12	2,36	0,25	-1,23	0,91611

	WT HFD		IEC MyD88-KO HFD			
	Mean	SEM	Mean	SEM	changes (%)	p-value
**Actinobacteria**	0,01	0,01	0,71	0,37	1727,84	0,08552
**Bacteroidetes**	29,25	2,95	34,90	2,77	19,30	0,18011
**Cyanobacteria**	0,03	0,01	0,03	0,02	131,95	0,55076
**Deferribacteres**	0,01	0,01	1,71	0,21	-12,17	0,59917
**Firmicutes**	0,04	0,01	50,99	2,27	-10,73	0,09286
**Proteobacteria**	1,94	0,39	8,56	0,80	-2,16	0,84616
**Tenericutes**	57,12	2,61	0,14	0,07	933,24	0,09244
**TM7**	8,75	0,54	0,07	0,04	99,46	0,44034
**unclassified**	2,36	0,25	0,51	0,05	7,32	0,60274
**unclassified Bacteria**	0,48	0,04	2,39	0,23	1,13	0,93842

All the phyla detected by pyrosequencing are presented in the tables. P-values are based on Student *t*-test.
Student *t*-test results were corrected by an FDR test according to the Benjamini-Hochberg procedure, with an α of < 0.05.

Supplementary Table 2: Relative abundance of the bacterial families found in the different groups (% of 16S rRNA sequences)

	WT		IEC MyD88-KO			
	**Mean**	**SEM**	**Mean**	**SEM**	**changes (%)**	**p-value**
Alcaligenaceae	1,528	0,731	1,627	0,521	6,47	0,91354
Anaeroplasmataceae	2,151	0,442	1,400	0,336	-34,89	0,19319
Bacteroidaceae	1,318	0,169	1,605	0,263	21,76	0,37042
Chloroplast	0,002	0,002	0,001	0,001	-42,48	0,65350
Clostridiaceae	0,013	0,010	0,003	0,002	-77,65	0,34955
Coriobacteriaceae	0,135	0,075	0,339	0,155	151,89	0,24962
Deferribacteraceae	5,617	0,845	5,249	0,895	-6,56	0,76811
Desulfovibrionaceae	5,230	0,649	4,662	0,619	-10,86	0,53435
Erysipelotrichaceae	3,900	1,131	4,465	0,487	14,47	0,65220
Incertae Sedis XIII	0,109	0,028	0,091	0,013	-16,16	0,58190
Incertae Sedis XIV	0,001	0,001	0,000	0,000	WT	0,33056
Lachnospiraceae	22,792	1,483	20,488	1,782	-10,11	0,33353
Lactobacillaceae	0,120	0,029	0,065	0,021	-46,32	0,14159
Porphyromonadaceae	11,702	1,239	14,866	1,216	27,03	0,08511
Prevotellaceae	0,016	0,014	0,000	0,000	WT	0,26360
Rikenellaceae	11,380	1,191	14,509	2,064	27,49	0,20578
Ruminococcaceae	18,036	1,377	13,974	0,779	-22,52	0,01940
Streptococcaceae	0,002	0,001	0,003	0,002	19,90	0,85406
TM7	0,070	0,015	0,057	0,014	-18,97	0,52643
u "Bacteroidales"	4,005	0,431	5,510	0,376	37,56	0,01702
u "Bacteroidetes"	1,519	0,226	1,733	0,133	14,06	0,42615
u "Clostridia"	0,362	0,057	0,242	0,050	-32,99	0,13418
u "Firmicutes"	1,958	0,147	1,722	0,234	-12,06	0,40432
u "Proteobacteria"	0,124	0,054	0,174	0,045	40,03	0,48661
u Alphaproteobacteria	0,247	0,113	0,320	0,082	29,58	0,60587
u Bacteria	2,392	0,120	2,099	0,178	-12,27	0,18891
u Burkholderiales	0,000	0,000	0,002	0,001	IEC MyD88-KO	0,15575
u Clostridiales	4,768	0,464	4,220	0,487	-11,50	0,42589
u Desulfovibrionales	0,001	0,001	0,003	0,002	226,32	0,27844
u Mollicutes	0,001	0,001	0,000	0,000	WT	0,16487
Unclassified	0,498	0,066	0,574	0,045	15,14	0,35580

FDR: non-significant.

	WT		WT HFD			
	**Mean**	**SEM**	**Mean**	**SEM**	**changes (%)**	**p-value**
Alcaligenaceae	1,528	0,731	0,063	0,051	-95,85	0,06092
Anaeroplasmataceae	2,151	0,442	0,013	0,007	-99,40	0,00013*
Bacteroidaceae	1,318	0,169	2,024	0,213	53,58	0,01819
Chloroplast	0,002	0,002	0,011	0,007	378,28	0,26689
Clostridiaceae	0,013	0,010	0,000	0,000	WT	0,22358
Coriobacteriaceae	0,135	0,075	0,039	0,012	-71,07	0,22517
Deferribacteraceae	5,617	0,845	1,943	0,390	-65,41	0,00094*
Desulfovibrionaceae	5,230	0,649	8,612	0,591	64,66	0,00116*
Enterobacteriaceae	0,000	0,000	0,007	0,003	WT HFD	0,02626
Erysipelotrichaceae	3,900	1,131	0,187	0,095	-95,21	0,00424*
Incertae Sedis XIII	0,109	0,028	0,041	0,005	-62,61	0,03006
Incertae Sedis XIV	0,001	0,001	0,001	0,001	-0,42	0,99765
Lachnospiraceae	22,792	1,483	25,612	2,457	12,37	0,33889
Lactobacillaceae	0,120	0,029	0,029	0,011	-75,75	0,00963*
Peptococcaceae	0,000	0,000	0,001	0,001	WT HFD	0,15293
Peptostreptococcaceae	0,000	0,000	0,035	0,012	WT HFD	0,00714*
Porphyromonadaceae	11,702	1,239	5,741	0,692	-50,94	0,00054*
Prevotellaceae	0,016	0,014	0,051	0,020	213,52	0,17635
Rikenellaceae	11,380	1,191	16,333	1,695	43,52	0,02796
Ruminococcaceae	18,036	1,377	23,710	1,238	31,46	0,00669*
Streptococcaceae	0,002	0,001	0,006	0,002	178,14	0,15706
TM7	0,070	0,015	0,033	0,010	-52,36	0,05468
u "Bacteroidales"	4,005	0,431	3,874	0,453	-3,29	0,83570
u "Bacteroidetes"	1,519	0,226	1,227	0,129	-19,22	0,27691
u "Clostridia"	0,362	0,057	0,126	0,024	-65,12	0,00130*
u "Firmicutes"	1,958	0,147	2,443	0,388	24,77	0,25767
u "Proteobacteria"	0,124	0,054	0,023	0,009	-81,90	0,07839
u Alphaproteobacteria	0,247	0,113	0,046	0,019	-81,36	0,09536
u Bacteria	2,392	0,120	2,363	0,248	-1,23	0,91607
u Burkholderiales	0,000	0,000	0,000	0,000	WT HFD	
u Clostridiales	4,768	0,464	4,925	0,280	3,29	0,77567
u Desulfovibrionales	0,001	0,001	0,001	0,001	31,51	0,81634
u Mollicutes	0,001	0,001	0,001	0,001	-41,90	0,64804
Unclassified	0,498	0,066	0,478	0,040	-4,14	0,79147

*: significant p-values after FDR correction.

	IEC MyD88-KO					
	WT HFD		HFD			
	Mean	SEM	Mean	SEM	changes (%)	p-value
Alcaligenaceae	0,06	0,05	0,12	0,06	84,85	0,51937
Anaeroplasmataceae	0,01	0,01	0,14	0,07	996,67	0,09163
Bacteroidaceae	2,02	0,21	3,23	0,52	59,84	0,04638
Chloroplast	0,01	0,01	0,03	0,02	131,95	0,55076
Clostridiaceae	0,00	0,00	0,01	0,00	IEC MyD88-KO HFD	0,05148
Coriobacteriaceae	0,04	0,01	0,71	0,37	1727,85	0,08552
Deferribacteraceae	1,94	0,39	1,71	0,21	-12,17	0,59918
Desulfovibrionaceae	8,61	0,59	8,37	0,79	-2,78	0,81155
Enterobacteriaceae	0,01	0,00	0,01	0,01	62,54	0,58657
Erysipelotrichaceae	0,19	0,10	1,12	0,33	500,20	0,01359
Incertae Sedis XIII	0,04	0,01	0,08	0,02	97,96	0,03597
Incertae Sedis XIV	0,00	0,00	0,00	0,00	WT HFD	0,33056
Lachnospiraceae	25,61	2,46	24,52	1,73	-4,25	0,72170
Lactobacillaceae	0,03	0,01	0,07	0,02	133,91	0,09718
Peptococcaceae	0,00	0,00	0,00	0,00	74,07	0,58792
Peptostreptococcaceae	0,04	0,01	0,12	0,06	250,81	0,17726
Porphyromonadaceae	5,74	0,69	7,01	0,59	22,19	0,17904
Prevotellaceae	0,05	0,02	0,03	0,01	-49,00	0,28999
Rikenellaceae	16,33	1,69	18,59	1,36	13,84	0,31235
Ruminococcaceae	23,71	1,24	19,12	1,50	-19,37	0,02992
Streptococcaceae	0,01	0,00	0,01	0,00	35,40	0,48599
TM7	0,03	0,01	0,07	0,04	99,46	0,44034
u "Bacteroidales"	3,87	0,45	4,76	0,43	22,76	0,17453
u "Bacteroidetes"	1,23	0,13	1,27	0,11	3,61	0,79786
u "Clostridia"	0,13	0,02	0,07	0,01	-48,09	0,02826
u "Firmicutes"	2,44	0,39	1,15	0,12	-52,75	0,00540
u "Proteobacteria"	0,02	0,01	0,03	0,01	34,50	0,56704
u Alphaproteobacteria	0,05	0,02	0,03	0,01	-34,00	0,49409
u Bacteria	2,36	0,25	2,39	0,23	1,13	0,93839
u Clostridiales	4,92	0,28	4,71	0,45	-4,37	0,68821
u Desulfovibrionales	0,00	0,00	0,00	0,00	15,20	0,88838
u Mollicutes	0,00	0,00	0,00	0,00	-17,79	0,89222
Unclassified	0,48	0,04	0,51	0,05	7,32	0,60271

All the families detected by pyrosequencing are presented in the tables. Indication WT, IEC MyD88-KO, WT HFD or IEC MyD88-KO HFD in the column ‘changes (%)’ corresponds to the group of mice for which the given phylotype was found exclusively. P-values are based on Student *t*-test.
u=unclassified
FDR: non-significant.

Supplementary Table 3: Relative abundance of bacterial genera found in the different groups (% of 16S rRNA sequences)

	WT		IEC MyD88-KO			
	Mean	SEM	Mean	SEM	changes (%)	p-value
Alistipes	11,374	1,192	14,505	2,064	27,52	0,20561
Allobaculum	3,292	0,957	3,812	0,421	15,80	0,62494
Anaeroplasma	2,151	0,442	1,400	0,336	-34,89	0,19319
Anaerostipes	0,001	0,001	0,000	0,000	WT	0,16487
Anaerotruncus	0,742	0,100	0,460	0,116	-38,08	0,08181
Anaerovorax	0,109	0,028	0,091	0,013	-16,16	0,58190
Asaccharobacter	0,013	0,006	0,008	0,004	-38,06	0,51239
Bacteroides	1,318	0,169	1,605	0,263	21,76	0,37042
Barnesiella	0,004	0,002	0,010	0,004	179,98	0,17593
Blautia	0,001	0,001	0,000	0,000	WT	0,33056
Butyricicoccus	0,046	0,007	0,033	0,008	-29,62	0,22342
Butyrivibrio	0,186	0,058	0,087	0,021	-53,13	0,12683
Clostridium	0,013	0,010	0,003	0,002	-77,65	0,34955
Coprococcus	0,055	0,008	0,027	0,004	-51,57	0,00687
Dorea	0,218	0,030	0,140	0,018	-35,64	0,03896
Enterorhabdus	0,000	0,000	0,001	0,001	IEC MyD88-KO	0,33056
Lactobacillus	0,120	0,029	0,065	0,021	-46,32	0,14159
Lactococcus	0,001	0,001	0,003	0,002	363,00	0,34235
Lactonifactor	0,001	0,001	0,000	0,000	WT	0,33056
Lawsonia	0,241	0,040	0,211	0,034	-12,26	0,57891
Marvinbryantia	0,025	0,011	0,004	0,003	-84,17	0,08241
Mucispirillum	5,617	0,845	5,249	0,895	-6,56	0,76811
Odoribacter	4,172	0,464	4,160	0,509	-0,29	0,98619
Olsenella	0,098	0,057	0,285	0,141	191,05	0,23413
Oscillibacter	10,097	0,982	7,779	0,394	-22,96	0,04194
Parabacteroides	0,281	0,044	0,255	0,037	-9,26	0,65742
Parasutterella	1,528	0,731	1,627	0,521	6,47	0,91354
Prevotella	0,015	0,013	0,000	0,000	WT	0,25929
Rikenella	0,001	0,001	0,000	0,000	WT	0,33056
Robinsoniella	0,001	0,001	0,000	0,000	WT	0,15335
Roseburia	0,003	0,001	0,001	0,001	-57,34	0,27252
Streptococcus	0,002	0,001	0,000	0,000	WT	0,15605
Streptophyta	0,002	0,002	0,001	0,001	-42,48	0,65350
Syntrophococcus	0,016	0,005	0,012	0,004	-25,19	0,53447
TM7_genera_incertae_sedis	0,070	0,015	0,057	0,014	-18,97	0,52643
u "Bacteroidales"	4,005	0,431	5,510	0,376	37,56	0,01702
u "Bacteroidetes"	1,519	0,226	1,733	0,133	14,06	0,42615
u "Clostridia"	0,362	0,057	0,242	0,050	-32,99	0,13418
u "Firmicutes"	1,958	0,147	1,722	0,234	-12,06	0,40432
u "Lachnospiraceae"	22,287	1,432	20,217	1,762	-9,29	0,37392
u "Porphyromonadaceae"	7,245	1,305	10,440	0,940	44,10	0,06238
u "Prevotellaceae"	0,001	0,001	0,000	0,000	WT	0,33056
u "Proteobacteria"	0,124	0,054	0,174	0,045	40,03	0,48661
u "Rikenellaceae"	0,005	0,002	0,004	0,002	-23,75	0,67833
u "Ruminococcaceae"	7,150	0,530	5,703	0,413	-20,23	0,04511
u Alphaproteobacteria	0,247	0,113	0,320	0,082	29,58	0,60587
u Bacteria	2,392	0,120	2,099	0,178	-12,27	0,18891
u Burkholderiales	0,000	0,000	0,002	0,001	IEC MyD88-KO	0,15575
u Clostridiales	4,768	0,464	4,220	0,487	-11,50	0,42589
u Coriobacteriaceae	0,024	0,016	0,046	0,016	89,13	0,35231
u Desulfovibrionaceae	4,989	0,615	4,451	0,587	-10,79	0,53441
u Desulfovibrionales	0,001	0,001	0,003	0,002	226,32	0,27844
u Erysipelotrichaceae	0,608	0,175	0,652	0,070	7,27	0,81745
u Mollicutes	0,001	0,001	0,000	0,000	WT	0,16487

Unclassified	0,498	0,066	0,574	0,045	15,14	0,35580
u "Bacteroidales"	4,005	0,431	5,510	0,376	37,56	0,01702

All the genera detected by pyrosequencing are presented in the tables. Indication WT, IEC MyD88-KO, WT HFD or IEC MyD88-KO HFD in the column ‘changes (%)’ corresponds to the group of mice for which the given phylotype was found exclusively. P-values are based on Student *t*-test.
u=unclassified. Significant P-values (≤0.05 are indicated in red).
FDR: non-significant.

	WT		WT HFD			
	Mean	SEM	Mean	SEM	changes (%)	p-value
Alistipes	11,374	1,192	16,329	1,694	43,56	0,02789
Allobaculum	3,292	0,957	0,151	0,079	-95,42	0,00425*
Anaeroplasma	2,151	0,442	0,013	0,007	-99,40	0,00013*
Anaerostipes	0,001	0,001	0,000	0,000	WT	0,16487
Anaerotruncus	0,742	0,100	0,437	0,047	-41,16	0,01259
Anaerovorax	0,109	0,028	0,041	0,005	-62,61	0,03006
Asaccharobacter	0,013	0,006	0,006	0,004	-50,78	0,37722
Bacteroides	1,318	0,169	2,024	0,213	53,58	0,01819
Barnesiella	0,004	0,002	0,000	0,000	WT	0,05274
Blautia	0,001	0,001	0,001	0,001	-0,42	0,99765
Butyricicoccus	0,046	0,007	0,089	0,010	90,81	0,00250*
Butyrivibrio	0,186	0,058	0,033	0,013	-82,18	0,01970
Clostridium	0,013	0,010	0,000	0,000	WT	0,22358
Coprococcus	0,055	0,008	0,038	0,010	-31,39	0,19430
Dorea	0,218	0,030	0,078	0,028	-64,16	0,00296*
Enterorhabdus	0,000	0,000	0,001	0,001	WT HFD	0,33056
Escherichia/Shigella	0,000	0,000	0,007	0,003	WT HFD	0,02626
Hydrogenoanaerobacterium	0,000	0,000	0,001	0,001	WT HFD	0,33056
Lactobacillus	0,120	0,029	0,029	0,011	-75,75	0,00963*
Lactococcus	0,001	0,001	0,005	0,002	865,21	0,05097
Lactonifactor	0,001	0,001	0,002	0,001	260,89	0,23772
Lawsonia	0,241	0,040	0,374	0,043	55,19	0,03570
Marvinbryantia	0,025	0,011	0,003	0,002	-89,83	0,05905
Mucispirillum	5,617	0,845	1,943	0,390	-65,41	0,00094*
Odoribacter	4,172	0,464	2,672	0,474	-35,96	0,03644
Olsenella	0,098	0,057	0,014	0,009	-85,96	0,16086
Oscillibacter	10,097	0,982	12,931	0,600	28,07	0,02415
Parabacteroides	0,281	0,044	0,245	0,048	-12,78	0,58903
Parasutterella	1,528	0,731	0,063	0,051	-95,85	0,06092
Prevotella	0,015	0,013	0,046	0,018	202,63	0,18335
Rikenella	0,001	0,001	0,000	0,000	WT	0,33056
Robinsoniella	0,001	0,001	0,000	0,000	WT	0,15335
Roseburia	0,003	0,001	0,000	0,000	WT	0,02822
Sporacetigenium	0,000	0,000	0,028	0,010	WT HFD	0,01161
Streptococcus	0,002	0,001	0,001	0,001	-61,95	0,43463
Streptophyta	0,002	0,002	0,011	0,007	378,28	0,26689
Syntrophococcus	0,016	0,005	0,010	0,004	-34,57	0,36835
TM7_genera_incertae_sedis	0,070	0,015	0,033	0,010	-52,36	0,05468
u "Bacteroidales"	4,005	0,431	3,874	0,453	-3,29	0,83570
u "Bacteroidetes"	1,519	0,226	1,227	0,129	-19,22	0,27691
u "Clostridia"	0,362	0,057	0,126	0,024	-65,12	0,00130*
u "Firmicutes"	1,958	0,147	2,443	0,388	24,77	0,25767
u "Lachnospiraceae"	22,287	1,432	25,450	2,416	14,19	0,27491
u "Porphyromonadaceae"	7,245	1,305	2,823	0,263	-61,03	0,00379*
u "Prevotellaceae"	0,001	0,001	0,005	0,003	377,64	0,21316
u "Proteobacteria"	0,124	0,054	0,023	0,009	-81,90	0,07839
u "Rikenellaceae"	0,005	0,002	0,003	0,002	-36,95	0,54513
u "Ruminococcaceae"	7,150	0,530	10,249	0,680	43,36	0,00207*
u Alphaproteobacteria	0,247	0,113	0,046	0,019	-81,36	0,09536
u Bacteria	2,392	0,120	2,363	0,248	-1,23	0,91607
u Clostridiales	4,768	0,464	4,925	0,280	3,29	0,77567
u Coriobacteriaceae	0,024	0,016	0,018	0,006	-24,38	0,73570
u Desulfovibrionaceae	4,989	0,615	8,239	0,554	65,12	0,00099*
u Desulfovibrionales	0,001	0,001	0,001	0,001	31,51	0,81634
u Erysipelotrichaceae	0,608	0,175	0,036	0,016	-94,06	0,00447*
u Mollicutes	0,001	0,001	0,001	0,001	-41,90	0,64804

Unclassified	0,498	0,066	0,478	0,040	-4,14	0,79147

All the genera detected by pyrosequencing are presented in the tables. Indication WT, IEC MyD88-KO, WT HFD or IEC MyD88-KO HFD in the column 'changes (%)' corresponds to the group of mice for which the given phylotype was found exclusively. P-values are based on Student *t*-test. u=unclassified. Significant P-values (≤0.05 are indicated in red).
*: significant p-values after FDR correction.

	IEC MyD88-KO					
	WT HFD		HFD			
	Mean	SEM	Mean	SEM	changes (%)	p-value
Alistipes	16,329	1,694	18,589	1,363	13,84	0,31245
Allobaculum	0,151	0,079	0,916	0,269	507,80	0,01373
Anaeroplasma	0,013	0,007	0,142	0,072	996,67	0,09163
Anaerotruncus	0,437	0,047	0,257	0,043	-41,26	0,01094
Anaerovorax	0,041	0,005	0,081	0,017	97,96	0,03597
Asaccharobacter	0,006	0,004	0,013	0,006	109,78	0,34893
Bacteroides	2,024	0,213	3,235	0,524	59,84	0,04638
Blautia	0,001	0,001	0,000	0,000	WT HFD	0,33056
Butyricicoccus	0,089	0,010	0,056	0,014	-36,70	0,06957
Butyrivibrio	0,033	0,013	0,026	0,008	-22,51	0,63422
Clostridium	0,000	0,000	0,010	0,005	IEC MyD88-KO HFD	0,05148
Coprococcus	0,038	0,010	0,024	0,007	-37,57	0,25336
Dorea	0,078	0,028	0,051	0,015	-34,71	0,39969
Enterorhabdus	0,001	0,001	0,000	0,000	WT HFD	0,33056
Escherichia/Shigella	0,007	0,003	0,011	0,007	62,54	0,58657
Hydrogenoanaerobacterium	0,001	0,001	0,000	0,000	WT HFD	0,33056
Lactobacillus	0,029	0,011	0,068	0,019	133,91	0,09718
Lactococcus	0,005	0,002	0,007	0,002	29,46	0,60137
Lactonifactor	0,002	0,001	0,002	0,002	-19,91	0,83743
Lawsonia	0,374	0,043	0,319	0,053	-14,69	0,42958
Marvinbryantia	0,003	0,002	0,000	0,000	WT HFD	0,16043
Mucispirillum	1,943	0,390	1,706	0,209	-12,17	0,59918
Odoribacter	2,672	0,474	3,382	0,340	26,57	0,23954
Olsenella	0,014	0,009	0,645	0,343	4593,30	0,08225
Oscillibacter	12,931	0,600	10,788	0,862	-16,57	0,05621
Parabacteroides	0,245	0,048	0,230	0,057	-6,20	0,84128
Parasutterella	0,063	0,051	0,117	0,064	84,85	0,51937
Prevotella	0,046	0,018	0,025	0,011	-45,20	0,33386
Sporacetigenium	0,028	0,010	0,102	0,052	261,55	0,17634
Streptococcus	0,001	0,001	0,001	0,001	88,12	0,68404
Streptophyta	0,011	0,007	0,026	0,023	131,95	0,55076
Syntrophococcus	0,010	0,004	0,009	0,004	-9,58	0,85312
TM7_genera_incertae_sedis	0,033	0,010	0,066	0,041	99,46	0,44034
u "Bacteroidales"	3,874	0,453	4,756	0,428	22,76	0,17453
u "Bacteroidetes"	1,227	0,129	1,271	0,111	3,61	0,79786
u "Clostridia"	0,126	0,024	0,066	0,009	-48,09	0,02826
u "Firmicutes"	2,443	0,388	1,154	0,124	-52,75	0,00540
u "Lachnospiraceae"	25,450	2,416	24,414	1,729	-4,07	0,73151
u "Porphyromonadaceae"	2,823	0,263	3,402	0,320	20,50	0,17933
u "Prevotellaceae"	0,005	0,003	0,001	0,001	-85,30	0,16828
u "Proteobacteria"	0,023	0,009	0,030	0,010	34,50	0,56704
u "Rikenellaceae"	0,003	0,002	0,004	0,002	21,44	0,80630
u "Ruminococcaceae"	10,249	0,680	8,015	0,742	-21,80	0,03947
u Alphaproteobacteria	0,046	0,019	0,030	0,012	-34,00	0,49409
u Bacteria	2,363	0,248	2,389	0,232	1,13	0,93839
u Clostridiales	4,925	0,280	4,710	0,447	-4,37	0,68821
u Coriobacteriaceae	0,018	0,006	0,054	0,023	196,91	0,15296
u Desulfovibrionaceae	8,239	0,554	8,054	0,763	-2,24	0,84701
u Desulfovibrionales	0,001	0,001	0,001	0,001	15,20	0,88838
u Erysipelotrichaceae	0,036	0,016	0,205	0,065	468,48	0,02080
u Mollicutes	0,001	0,001	0,001	0,001	-17,79	0,89222
Unclassified	0,478	0,040	0,513	0,052	7,32	0,60271

All the genera detected by pyrosequencing are presented in the tables. Indication WT, IEC MyD88-KO, WT HFD or IEC MyD88-KO HFD in the column ‘changes (%)’ corresponds to the group of mice for which the given phylotype was found exclusively. P-values are based on Student *t*-test.
u=unclassified. Significant P-values (≤0.05 are indicated in red).
FDR: non-significant.

Supplementary Table 4: Primers sequences

Primers	Forward Sequence	Reverse Sequence
RPL-19	GAAGGTCAAAGGGAATGTGTTCA	CCTGTTGCTCACTTGT
CD11c	ACGTCAGTACAAGGAGATGTTGGA	ATCCTATTGCAGAATGCTTCTTTACC
MCP1	GCAGTTAACGCCCCACTCA	CCCAGCCTACTCATTGGGATCA
CB1	CTGATGTTCTGGATCGGAGTC	TCTGAGGTGTGAATGATGATGC
GPR119	AGCTCTGCTCAGCACACACAG	GAATGCCATCCGAAGGCTAC
Reg3g	TTCCTGTCCTCCATGATCAAA	CATCCACCTCTGTTGGGTTC
Lyz1	GCCAAGGTCTACAATCGTTGTGAGTTG	CAGTCAGCCAGCTTGACACCACG
Pla2g2a	AGGATTCCCCCAAGGATGCCAC	CAGCCGTTTCTGACAGGAGTTCTGG
Defa	GGTGATCATCAGACCCCAGCATCAGT	AAGAGACTAAAACTGAGGAGCAGC
Ang 4	CTCTGGCTCAGAATGTAAGGTACGA	GAAATCTTTAAAGGCTCGGTACCC
Foxp3	TCCTTCCCAGAGTTCTTCCA	CGAACATGCGAGTAAACCAA
Myd88	CGGAACTTTTCGATGCCTTT	TAGTTGCCGGATCATCTCCT
PPARg	CTGCTCAAGTATGGTGTCCATGA	TGAGATGAGGACTCCATCTTTATTCA
FIAF	CAATGCCAAATTGCTCCAATT	TGGCCGTGGGCTCAGT
IL18	ACAACTTTGGCCGACTTCAC	CTGGGGTTCACTGGCACTTT
IL1b	TCGCTCAGGGTCACAAGAAA	CATCAGAGGCAAGGAGGAAAAC
TRAF	TGGCATTCCTACCATCCTTT	TCGGAAGGCTTCATATGCTC
Tjp1	TTTTTGACAGGGGGAGTGG	TGCTGCAGAGGTCAAAGTTCAAG
Claudin2	AAGGTGCTGCTGAGGGTAGA	AGTGGCAGAGATGGGATTTG
Occludin	ATGTCCGGCCGATGCTCTC	TTTGGCTGCTCTTGGGTCTGTAT
